# A general strategy exploiting m^5^C duplex-remodelling effect for selective detection of RNA and DNA m^5^C methyltransferase activity in cells

**DOI:** 10.1093/nar/gkz1047

**Published:** 2019-11-06

**Authors:** Tianming Yang, Joanne J A Low, Esther C Y Woon

**Affiliations:** Department of Pharmacy, National University of Singapore, 18 Science Drive 4, 117543 Singapore

## Abstract

RNA:5-methylcytosine (m^5^C) methyltransferases are currently the focus of intense research following a series of high-profile reports documenting their physiological links to several diseases. However, no methods exist which permit the specific analysis of RNA:m^5^C methyltransferases in cells. Herein, we described how a combination of biophysical studies led us to identify distinct duplex-remodelling effects of m^5^C on RNA and DNA duplexes. Specifically, m^5^C induces a C3′-*endo* to C2′-*endo* sugar-pucker switch in CpG RNA duplex but triggers a B-to-Z transformation in CpG DNA duplex. Inspired by these different ‘structural signatures’, we developed a m^5^C-sensitive probe which fluoresces spontaneously in response to m^5^C-induced sugar-pucker switch, hence useful for sensing RNA:m^5^C methyltransferase activity. Through the use of this probe, we achieved real-time imaging and flow cytometry analysis of NOP2/Sun RNA methyltransferase 2 (NSUN2) activity in HeLa cells. We further applied the probe to the cell-based screening of NSUN2 inhibitors. The developed strategy could also be adapted for the detection of DNA:m^5^C methyltransferases. This was demonstrated by the development of DNA m^5^C-probe which permits the screening of DNA methyltransferase 3A inhibitors. To our knowledge, this study represents not only the first examples of m^5^C-responsive probes, but also a new strategy for discriminating RNA and DNA m^5^C methyltransferase activity in cells.

## INTRODUCTION

The DNA and RNA of all living organisms, as well as that of viruses, mitochondria and chloroplasts, undergo a wide range of modifications (1–2). These modifications not only expand the structural diversity of nucleic acids, but also provide an epigenetic mechanism to fine-tune their biological functions (3–4). To date, at least 160 naturally-occurring chemical modifications have been identified, amongst which 5-methylcytosine (m^5^C) is currently one of the most intensively studied epigenetic modifications. The m^5^C modification is prevalent in DNA and multiple RNA classes (5–6). Its biological functions are best understood in DNA, where it is involved in the regulation of gene expression, genome reprogramming, organismal development and cellular differentiation (5,7,8). In contrast, there has been comparatively less studies on the biological roles of m^5^C in RNA. Indeed the significance of m^5^C modification in mRNA was not fully appreciated until recently, following the landmark discovery of widespread m^5^C sites in the transcriptomes of diverse organisms (9–16), suggesting that the m^5^C modification is far more pervasive in human mRNA than previously realised. This has reignited intense interest in the study of this epitranscriptomic mark. At present, the exact biological function of m^5^C modification in mRNA remains elusive although it has been linked to many cellular processes, such as nuclear export regulation (14), modulation of protein translation (17–19), and stress response (20).

The m^5^C methylation landscape is regulated by a complex array of m^5^C methyltransferases (MTases) and m^5^C demethylases, which specifically add and remove a 5-methyl mark from cytosine base, respectively (6,21–23). In humans, C-5 cytosine methylation of DNA is catalysed by at least three DNA:m^5^C MTases (*i.e*. DNMT1, 3A and 3B), whereas m^5^C methylation of RNA is catalysed by the NOL1/NOP2/Sun domain (NSUN) RNA methyltransferase family, which includes NSUN1-7, as well as the DNA MTase homologue TRDMT1 (formerly DNMT2). Notably, both the DNA and RNA m^5^C MTases employ a common *S*-adenosyl-l-methionine (SAM) dependent mechanism for methyl group transfer, although different active-site cysteine residues are likely involved in the catalysis (24).

In recent years, it has become increasingly clear that aberrant expression of m^5^C MTases may underlie the pathogenesis of several diseases. For instance, DNMT3A, NSUN1, NSUN2 and NSUN4 were found to be overexpressed in a number of human cancers, including breast, prostate, cervical, and colorectal cancers (25–29). NSUN3 is linked to mitochondrial diseases (30,31), whilst NSUN5 and NSUN7 are likely implicated in Williams-Beuren syndrome (a neurodevelopmental disorder) (32) and male infertility (33), respectively. Given the strong physiological links, there is currently immense interest in studying the biological roles of these enzymes and their potentials as therapeutic targets. However, at present, no methods exist which permit the direct analysis of specific m^5^C MTase in living cells. There are also no reports of cell-based assays capable of discriminating between DNA and RNA MTase activities. This severely impedes our understanding of these medically-important enzymes.

It is known that several nucleic acid base methylations can directly impact the secondary structure of DNA and RNA. For instance, we (34–36), and others (37–40), have recently shown that the presence of a single *N*^6^-methyladenosine (m^6^A) or *N*^1^-methyladenosine (m^1^A) modification can trigger a duplex-hairpin transformation in certain RNA sequence contexts. It is also clear from a number of NMR and X-ray crystallographic studies that m^5^C modification is able to induce a major B–Z structural change in some DNA sequences. This phenomenon was first reported by Behe and Felsenfeld (41), and subsequently by others (42–44), for a number of DNA duplexes with alternating CpG sequences, such as d(m^5^CGCGm^5^CG), poly d(m^5^CG)_*n*_ and poly d(Gm^5^C)_*n*_. The structural influence of m^5^C on RNA duplexes, however, is significantly less-studied. One early study on a poly(CG) RNA sequence suggests that m^5^C methylation likely elicit a conformational change from an A-type duplex to an atypical duplex structure (45), whereas a separate study using a different sequence context indicates no significant structural alteration (46). To the best of our knowledge, these two reports provide the only experimental data available in this regard and, to date, it remains unclear whether m^5^C modification has any major impact on the secondary structures of RNA duplexes.

Herein we demonstrated, through a combination of NMR, circular dichroism (CD), and thermodynamic studies, that m^5^C has different conformational effects on DNA and RNA duplexes, even for duplexes with identical CpG sequence. In the case of CpG RNA duplex, it induces a considerable distortion of the phosphate backbone and a C3′-*endo* to C2′-*endo* sugar pucker switch in the terminal residues whereas, in the case of CpG DNA duplex, m^5^C triggered a remarkable B-to-Z structural transformation. m^5^C therefore produces distinctly different ‘structural signatures’ on CpG RNA and DNA duplexes under the same physiologically-relevant conditions.

**Figure 1.**
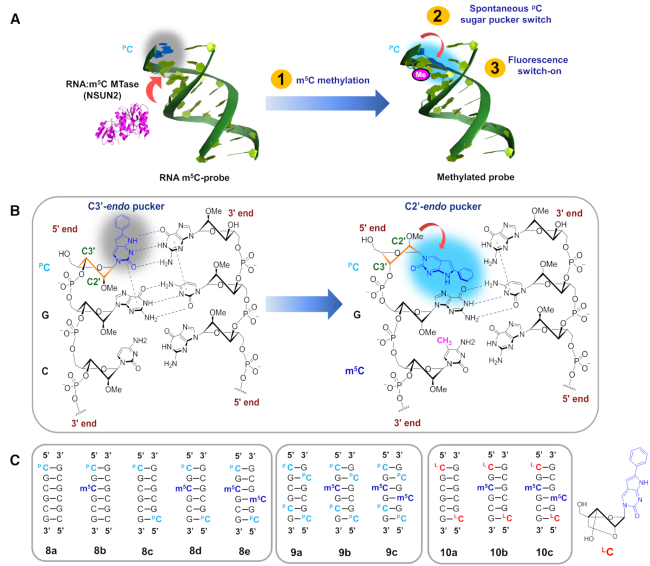
The m^5^C-switchable probe strategy. (**A**) The m^5^C-probe **8a** contains a 5′-terminal fluorescent nucleotide ^P^C (2′-*O*-methyl 6-phenylpyrrolocytidine), which lights up spontaneously in response to m^5^C-induced terminal sugar pucker switch. (**B**) When the probe is unmethylated, ^p^C is able to base-pair with guanine in the complementary strand and stack strongly with its adjacent base. This results in efficient quenching of ^p^C fluorescence through photoinduced electron transfer. m^5^C methylation of the probe by RNA:m^5^C MTase (e.g. NSUN2), however, is expected to trigger a C2′-*endo* to C3′-*endo* sugar pucker switch in ^P^C and, since the sugar ring pucker defines the glycosidic bond angle, such a change in sugar puckering will also convert the orientation of ^P^C base from axial to equatorial. This, in turn, disrupts its base-pairing and base-stacking interactions, leading to fluorescence activation. (**C**) Schematic representation of 2′-OMe RNA probes and their methylated counterparts. The composition of the probes was confirmed through MALDI-TOF mass spectrometric analysis (see Supplementary Table S2). The structure of ‘locked 6-phenylpyrrolocytidine’ (^L^C) is shown. The C2′−C4′ covalent link is geometrically incompatible with C2′-*endo* pucker mode, it therefore locks the nucleotide into the C3′-*endo* conformation.

Inspired by these interesting findings, we envisioned that the different duplex-remodelling effects of m^5^C could provide a basis for the selective detection of RNA and DNA m^5^C MTase activity in cells. This was demonstrated by the development of a novel m^5^C-sensitive nucleic acid probe which, by design, is capable of switching its terminal sugar pucker conformation spontaneously in response to m^5^C methylation (Figure 1). When coupled with an environment-sensitive fluorophore, it provides a powerful visual tool for sensing RNA:m^5^C methylation changes in cells. Prior to this work, we are not aware of any assay methods which are based on m^5^C-induced conformational change.

As we shall demonstrate, the m^5^C-probe is highly-selective and could specifically target NSUN2 over other RNA:m^5^C MTases (including structurally-related subfamily members NSUN3, NSUN5A, NSUN6) and DNA:m^5^C MTases (including DNMT1 and DNMT3A). Through the m^5^C-probe approach, we achieved live cell imaging and flow cytometry analyses of NSUN2 activity in HeLa cells. We also successfully applied the probe to the high-throughput cell-based screening of NSUN2 inhibitors. The discovery of such highly selective probes is rarely achieved and may provide insights on the functions of NSUN2 in m^5^C-regulated processes. Although this study focus on the sensing of RNA:m^5^C MTase activity, the m^5^C-switchable probe strategy outlined here could also be adapted for the study of DNA:m^5^C MTase. This was demonstrated by the successful development of DNA m^5^C-probe which is useful for the *in vitro* screening of DNMT3A inhibitors.

To the best of our knowledge, this study represents not only the first examples of m^5^C-responsive fluorescent probes, but also a new strategy for the discrimination of RNA and DNA m^5^C MTases activity in cells, which hopefully will inspire the development of epigenetic biosensors and diagnostics tools.

## MATERIALS AND METHODS

### Preparation of m^5^C-probes

All oligonucleotide probes investigated in this study were synthesized using standard β-cyanoethyl phosphoramidite chemistry on an automated DNA/RNA synthesiser (Applied Biosystems 394). All synthesiser reagents were purchased from Glen Research; 2′-*O-*methyl 6-phenylpyrrolocytidine phosphoramidite (47,48) and pyrene deoxynucleoside phosphoramidite (49) were synthesised according to literature procedure. For details of synthesis, see Supplementary Data. All oligonucleotides have a purity of at least 95% (Supplementary Tables S1-S3).

### Studies on the duplex-remodelling effects of m^5^C using NMR, CD and UV-based thermodynamic analyses

All ^1^H NMR experiments were recorded on a Bruker DRX-500 spectrometer operating at 500.23 MHz and the data processed using the standard Bruker processing software TopSpin. Estimated accuracy for protons is within 0.02 ppm. The oligos were dissolved in either D_2_O (99.98%) or buffer/D_2_O mix; the buffer used was 10 mM sodium phosphate buffer (pH 7.4) containing 150 mM NaCl and 20 mM MgCl_2_. Final concentrations were 3 mM for the unmethylated duplexes (**1a**, **7a**) and 2 mM for the m^5^C-methylated duplexes (**1b**, **7b**). The proton chemical shifts were referenced relative to residual H_2_O signal (4.75 ppm at 25°C). The ^1^H imino spectra of duplexes in 9:1 buffer/D_2_O solvent mix (pH 7.4) were acquired at 4°C and 10°C with suppression of water signal by gradient-tailored excitation (WATERGATE) sequence, as previously described (34,50); at these temperatures, the oligos exist in the duplex state, as verified by UV-melting and CD analyses. Two-dimensional ^1^H NMR spectra of duplexes in D_2_O and buffer/D_2_O were acquired in the phase-sensitive mode using previously described method (51). Two-dimensional NOESY spectra of duplexes in D_2_O (52–54) were recorded at 25°C with a spectral width of 4000 Hz using 1000 t_2_ complex points, 1000 t_1_ (real) points, pulse repetition delay of 2.5 s and mixing times (τ_m_) of 80, 150 and 300 ms. Two-dimensional NOESY spectra of duplexes in 9:1 buffer/D_2_O were acquired at 10°C with water suppression by WATERGATE sequence (34,50). Spectra were recorded using 2048 t_2_ complex points, 256 t_1_ (real) points, pulse repetition delay of 1.7 s and mixing time of 200 ms. Proton-decoupled ^31^P NMR spectra of duplexes in 9:1 buffer/D_2_O were acquired at 10°C on a Bruker AV300 spectrometer equipped with an autotune 5 mm QNP probe. Spectra were recorded at 121.4 MHz, with a spectral width of 10 kHz. The ^31^P chemical shifts were externally referenced to 85% phosphoric acid (H_3_PO_4_). Estimated accuracy for phosphorus is within 0.03 ppm. For CD spectroscopy and UV-based thermal denaturation experiments, see Supplementary Data.

### Fluorescence analyses of m^5^C-probes

Fluorescence analysis was performed using a Cary Eclipse fluorescence spectrophotometer, as previously reported with modifications (36). The fluorescence emission spectra (λ_em_ = 400–600 nm) of the probes were recorded in a quartz cuvette at 37°C, at a total stand concentration 5 μM in a buffer of 10 mM sodium phosphate buffer (pH 7.4) containing 150 mM NaCl and 20 mM MgCl_2_. An average of five scans was recorded. Background fluorescence spectra were acquired after the probe has been incubated for 30 min at 37°C. For determination of fluorescence quantum yields of the m^5^C-probes, see Supplementary Data.

### *In vitro* m^5^C-probe methyltransferase assay

*RNA methyltransferase assay:* the assay was performed at 37°C in triplicate in a Corning Costar 96-well flat bottom black plate. Reaction consisted of enzyme (0.5 μM), methyl donor *S-*adenosyl-l-methionine (SAM; 200 μM), m^5^C-probe **9a** (substrate; 5 μM) or locked-probe **10a** (control; 5 μM) in a 50 mM HEPES buffer (pH 7.4) containing 150 mM NaCl and 20 mM MgCl_2_. The final reaction volume is 25 μl. The time course of fluorescence activation was recorded immediately after the addition of enzyme with a Tecan ultra microplate reader (λ_ex_ 360 nm; λ_em_ 465 nm). An average of five scans was recorded. *DNA methyltransferase assay:* the assay was performed as described above. Reaction consisted of enzyme (NSUN2 or DNMT3A; 0.5 μM), methyl donor *S-*adenosyl-l-methionine (SAM; 200 μM), DNA m^5^C-probe **11a** (substrate; 5 μM) in a 50 mM HEPES buffer (pH 7.4) containing 150 mM NaCl and 20 mM MgCl_2_. The fluorescence of the reaction was monitored at λ_em_ 400 nm (λ_ex_ 340 nm).

### Steady-state kinetic analyses of the methylation of m^5^C-probe, tRNA and ssRNA by NSUN2

The substrates investigated include m^5^C-probe **9a**, human tRNA^Leu^(CAA) (5′-CCAGACUCAAGUUCUGG-3′), and CpG-rich ssRNA (5′-CGCGCGCGCGCG-3′). The kinetic constants (*V*_max_, *K*_m,_*k*_cat_) of the substrate were determined using an NSUN2 concentration of 0.1 μM and substrate concentrations of 1, 2, 3, 5, 8 and 10 μM. The amount of m^5^C-methylated product formed at different substrate concentrations was determined based on the relative intensities of the substrate and the product peaks observed in MALDI-TOF mass spectra. All reactions were performed at 37°C in triplicate.

### Fluorescence microscopy

The HeLa cells were grown in 35 mm glass bottom culture dishes (Thermo Scientific). The cells were transfected with either m^5^C-probe **9a** (10 μM) or locked-probe **10a** (10 μM; control) using Lipofectamine 2000 (Invitrogen) following the manufacturer's protocols. Fluorescence images were then acquired at 37°C in the presence of 5% CO_2_ using a Nikon BioStation IM-Q live cell imaging system equipped with 20× and 63× oil immersion objective lens. The fluorescence images were captured using the DAPI filter setting (i.e. λ_ex_ 340–380 nm; λ_em_ 435–485 nm) and the fluorescence intensity quantified using Biostation IM multichannel software by collecting mean gray values for regions exhibiting fluorescence. All experiments were performed in triplicates and the mean fluorescence intensities were normalised to that of the control. The corresponding transmitted light image for each fluorescence image was also acquired.

### Single-cell flow cytometry analysis

The HeLa cells were transfected with m^5^C-probe **9a** (10 μM) or control probe **10a** (10 μM) using Lipofectamine 2000. They were then collected by trypsinization at 37°C with 5% CO_2_, washed with phosphate buffered saline (PBS) media, pH 7.4, resuspended in PBS media, and analysed using a BD LSRFortessa™ flow cytometer (BD Bioscience). Fluorescence was measured using an excitation laser of 355 nm, and a 450/50 bandpass emission filter. Acquisition was stopped when 20, 000 events per sample were acquired. The fluorescence data were then analysed using BD FACS software. Violin plot was produced using the Origin software.

### Cell-based m^5^C-probe methyltransferase assay

The HeLa cells were pre-incubation with various concentrations of non-specific MTase inhibitors, including sinefungin, *S*-adenosyl-l-homocysteine (SAH), adenosine, and homocysteine at 37°C for 30 min. Nine different concentrations of inhibitors were used (0.1, 0.3, 1, 3, 10, 50, 100, 250, 1000 μM) and the final DMSO concentration was kept at less than 1% (v/v) of the assay mix. These concentrations of inhibitors were shown to be non-toxic to HeLa cells in MTT cytotoxicity assays. This was followed by the delivery of either the m^5^C-probe **9a** (10 μM) or control probe **10a** (10 μM) into the cells *via* Lipofectamine 2000. Aliquots were then collected for flow cytometry measurements (λ_ex_ 355 nm; λ_em_ 425–475 nm) at the indicated time points. The mean fluorescence intensity of at least 20 000 live cells was determined. *IC_50_ determination:* the IC_50_ values were then calculated from the variation in fluorescence at different inhibitor concentrations using nonlinear regression, with normalised dose-response fit on GraphPad Prism 6.0™. The assay was performed in triplicate for each inhibitor concentration. *Z’ factor determination:* the Z’ factor for our m^5^C-probe assay was calculated using previously described method (55). For details, see Supplementary Data.

### Cell culture, gene silencing and gene overexpression

HeLa cells (ATCC) were cultured in Dulbecco's modified Eagle's medium (DMEM; Life Technologies) supplemented with 10% fetal bovine serum (FBS) and antibiotics (100 units/ml penicillin, 100 μg/ml streptomycin). Cells were grown at 37°C in a 5% CO_2_ humidified incubator. To silence the expression of specific gene transiently, the cells were first plated in a 24-well plate (0.5 × 10^5^ cells/well). The next day, the cells were transfected with either siNSUN2, siNSUN6, siTRDMT1, siDNMT3A or siControl using Lipofectamine RNAiMAX (Invitrogen), according to manufacturer's protocols. The sequences used for siRNAs and siControls are provided in the Supplementary Data. The protein levels were analysed by western blot using specific antibodies. Overexpression of specific gene. The construction of pcDNA3-Flag vectors expressing NSUN2, TRDMT1 and DNMT3A was previously described (56–58). Full length *NSUN2, TRDMT1* and *DNMT3A* genes were amplified by PCR using human HeLa cells cDNA, then subcloned into the EcoRI-BamHI sites of mammalian pcDNA3-Flag vector (Invitrogen) to obtain pcDNA3-Flag-NSUN2, pcDNA3-Flag-TRDMT1, and pcDNA3-Flag-DNMT3A plasmids, respectively. The plasmids were then transfected into HeLa cells using the calcium chloride transfection method. For construction of pcDNA3-Flag-NSUN6 plasmid (59), the cDNA encoding *NSUN6* was PCR-amplified using Phusion DNA polymerase and subcloned into the KpnI-XhoI sites of pcDNA3.1-Flag with CloneExpress II One step Cloning Kit (Vazyme). The primer sequences used for vector construction are given in Supplementary Data.

### Western blot analysis

The HeLa cells were first lysed using radioimmunoprecipitation assay (RIPA) buffer (i.e. 25 mM Tris–HCl (pH 7.6), 150 mM NaCl, 0.1% SDS, 1% sodium deoxycholate, 1% NP-40 and 1× protease inhibitors), followed by immunoblotting using standard protocol. In brief, the extract was fractionated by sodium dodecyl sulfate-polyacrylamide gel electrophoresis (SDS-PAGE), transferred onto nitrocellulose filter membranes (Whatman), then incubation with the respective antibodies. This was followed by incubation with either horseradish peroxidase (HRP)-conjugated anti-rabbit IgG antibody (Bio-Rad) or HRP-conjugated anti-mouse IgG antibody (Bio-Rad) for 2 h at room temperature. Enhanced chemiluminescence substrates (Luminata Crescendo, EMD Millipore) were then applied and the signals exposed to autoradiography film. The immunoblots were then quantified by densitometric analyses using ImageJ software. All antibodies used were purchased from commercial sources (for details, see Supplementary Data).

## RESULTS AND DISCUSSION

### m^5^C induces a local distortion of the phosphate backbone and a C3′-*endo* to C2′-*endo* sugar pucker switch in CpG RNA duplex

To date, there has not been a systematic study directly comparing the structural impact of m^5^C methylation on CpG RNA duplexes and CpG DNA duplexes. Such an analysis might provide insights into the respective conformational and/or mechanistic roles of m^5^C in DNA and RNA, hence is of special interest. To investigate this question in multiple sequence contexts, we prepared a set of 6-mer and 12-mer RNA duplexes containing either alternating CpG sequences (**1a–2a**), out-of-alternation CpG sequence **3a** or random sequences (**4a–6a**), as well as their corresponding m^5^C-methylated counterparts (**1b**–**6b**; Table 1). To facilitate comparison, the CpG DNA duplexes **7a** and **7b** were also generated. For details of their chemical synthesis, see Supplementary Data.

**Table 1.** Sequences of RNA and DNA duplexes investigated in this study. The ^3^*J*_H1′−H2′_ values for the 5′- and 3′-terminal residues of each sequence are indicated in red and green, respectively. m^5^C-induced terminal sugar pucker switch was observed in 6-mer and 12-mer CpG duplexes (**1b** and **2b**) but not in out-of-alternation CpG duplex **3b** and random duplexes **4b–6b**, suggesting that the structural effect of m^5^C may be unique to alternating CpG RNA sequences. m^5^C methylation of **7a** i.e. the DNA equivalent of **1a** caused a C2′-*endo* to C3′-*endo* sugar pucker switch in all guanosine residues, whilst all cytosine residues preserved their C2′-*endo* orientation, consistent with a B-Z structural conversion

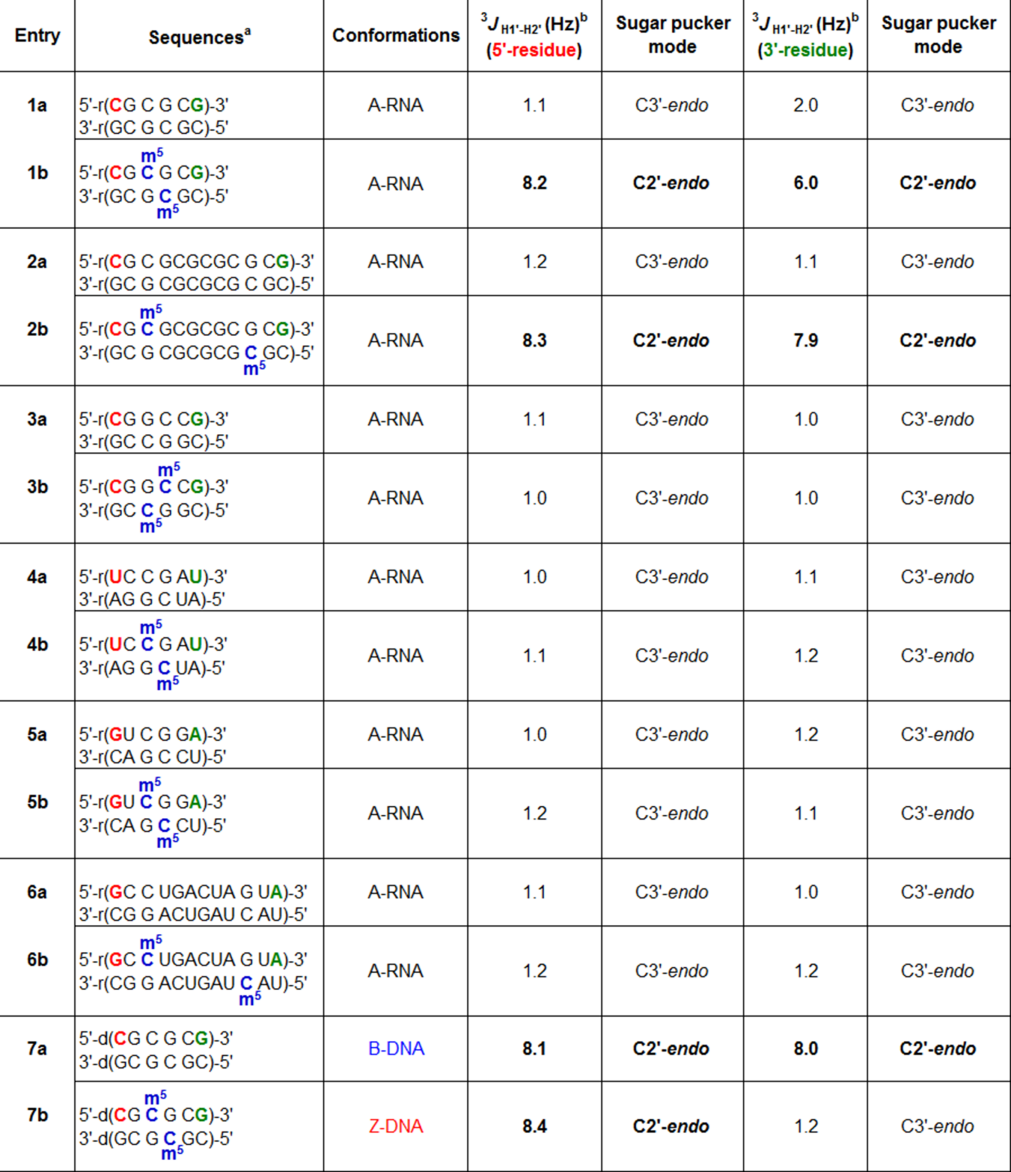

^a^The composition of the oligos was confirmed through MALDI-TOF mass spectrometric analysis (see Supplementary Table S1).

^b^Observed at 25°C in D_2_O.

**Figure 2.**
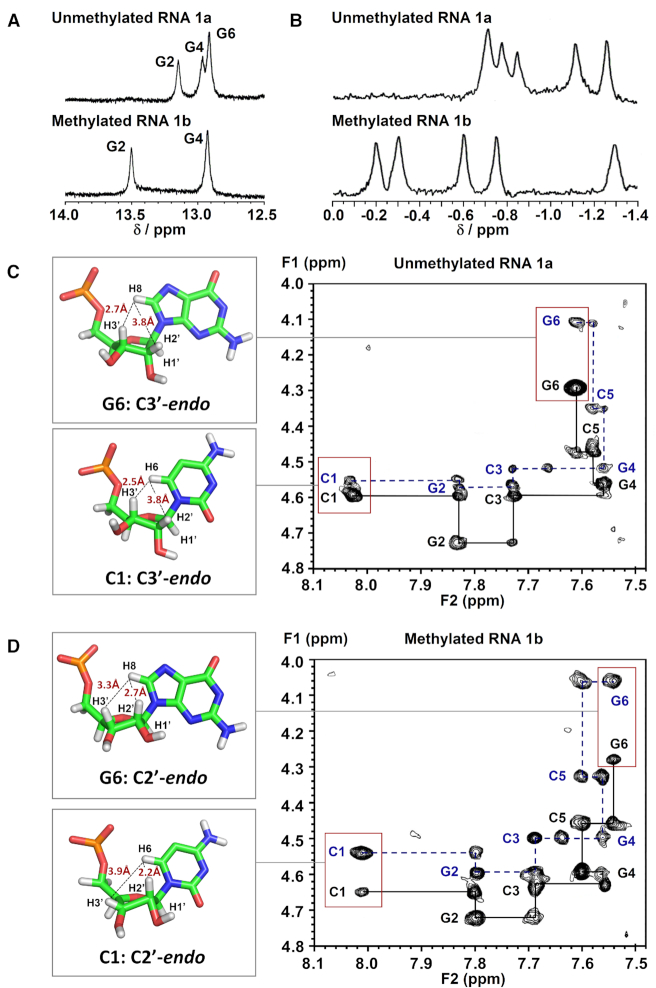
m^5^C methylation directly alters the phosphate backbone in CpG RNA duplex, leading to a C3′-*endo* to C2′-*endo* sugar pucker switch of the terminal residues under physiologically-relevant conditions. (**A**) Representative ^1^H imino NMR spectra of r(CG-C-GCG)_2_**1a** (top) and its methylated counterpart r(CG-**m^5^C**-GCG)_2_**1b** (bottom) in 9:1 buffer/D_2_O solvent mix acquired at 10°C. The buffer used was 10 mM sodium phosphate buffer (pH 7.4) containing 150 mM NaCl and 20 mM MgCl_2_. The G6 imino resonance disappeared upon m^5^C methylation, presumably due to ‘fraying’ of the terminal base-pairs. (**B**) Proton-decoupled ^31^P NMR spectra of **1a** (top) and **1b** (bottom) in 9:1 buffer/D_2_O solvent mix acquired at 10°C. There is considerable variations in ^31^P resonances between the methylated and unmethylated duplexes, suggesting a significant distortion of the phosphate backbone. Representative 2D NOESY spectra of the H2′/H3′-aromatic region of (**C**) **1a** and (**D**) **1b** in D_2_O at 25°C (pH 7.4; τ_m_ = 150 ms) showing the H6/8-H2′ NOE connectivity pathways (blue dashed-line) and the H6/8-H3′ NOE connectivity pathways (black solid line). The schematic illustrations (left) showed the likely sugar pucker mode for terminal residues C1 and G6, and their calculated intranucleotide base-sugar distances (labelled in red; determined based on the intensity of NOEs between the base and sugar protons).

We began by analysing the secondary structure of CpG RNA hexamer r(CG-C-GCG)_2_**1a** and its methylated equivalent r(CG-m^5^C-GCG)_2_**1b** using ^1^H and ^31^P NMR. Assignment of the proton resonances was accomplished by 2D NOESY experiments and by comparison with reported NMR data for **1a** (60). The results are summarised in Figure 2 and Table 2. Consistent with previous NMR studies (60), the imino proton spectrum of **1a** exhibited three NH signals at 10°C, which is expected for a palindromic duplex with six base-pairs (Figure 2A). Upon m^5^C methylation (**1b**), we observed a downfield shift in G2 resonance by ∼0.35 ppm and a disappearance of the terminal G6 resonance. The latter is likely due to ‘fraying’ of the terminal base-pairs as the G6 signal could again be detected at a lower temperature of 4°C (Supplementary Figure S1). This result suggests that m^5^C modification likely promotes the disruption of base-pairing interactions at duplex terminal ends. As anticipated, there is little or no change in the imino resonance of G4 which is involved in base-pairing with m^5^C, confirming that C5-cytosine methylation does not hinder C:G Watson–Crick base-pairing interactions. Nevertheless, we did observe considerable variations in ^31^P resonances between the methylated and unmethylated duplexes, indicating a significant distortion of the phosphate backbone (Figure 2B).

**Table 2.** ^1^H NMR chemical shifts δ_H_ (ppm) for CpG RNA duplex **1a** (top) and its m^5^C-methylated counterpart **1b** (bottom) observed at 25°C in D_2_O with WATERGATE suppression (τ_m_ 150 ms). Assignment of the proton resonances was accomplished by 2D NOESY experiments, and by comparison with reported NMR data for **1a** (60). The representative NMR spectra for the determination of ^3^*J*_H1′−H2′_ coupling constants are given in Supplementary Figure S2. For calculation of sugar-base proton distances, see Supplementary Data

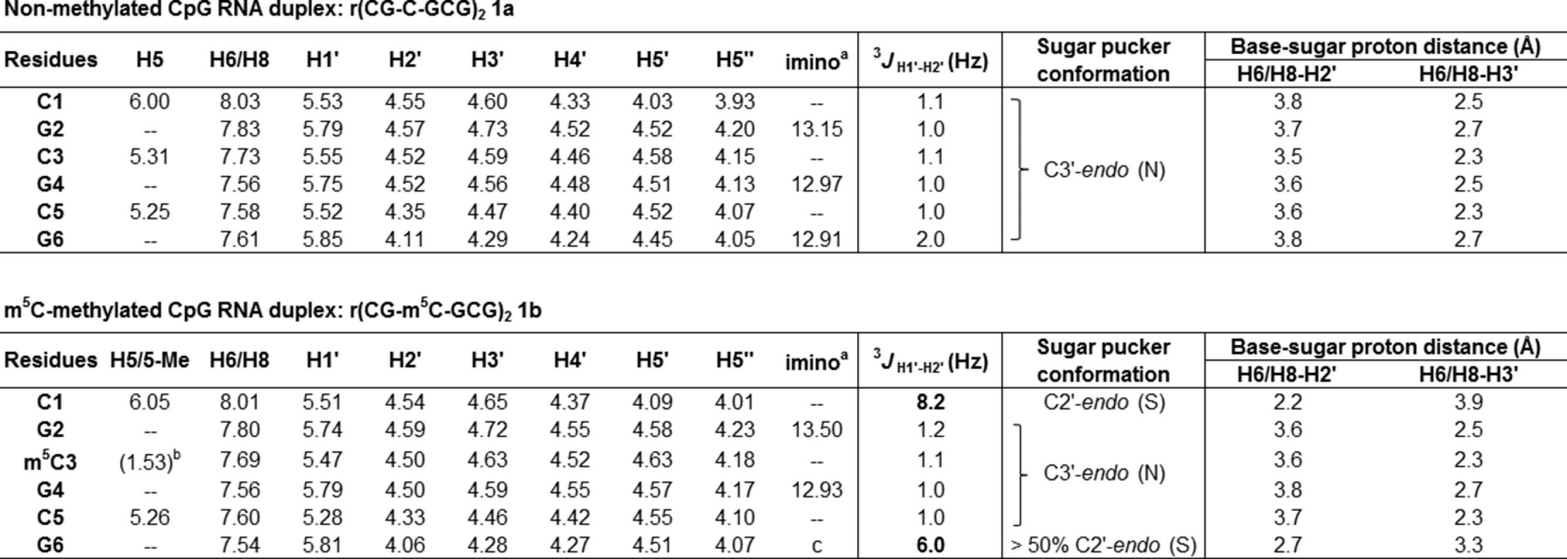

^a^Observed at 10°C in 9:1 buffer/D_2_O solvent mix. The buffer used was 10 mM sodium phosphate buffer (pH 7.4) containing 150 mM NaCl and 20 mM MgCl_2_.

^b^Chemical shift for 5-methyl group on m^5^C.

^c^Not observed even at 4°C.

Prompted by this finding, we proceeded to examine whether changes in phosphate backbone also alter the sugar ring geometry. To this end, we determined the coupling constant between sugar proton H1′ and H2*′* (^3^*J*_H1′−H2′_), which is highly sensitive to the dihedral angle and, therefore, provides a direct indication of the sugar pucker conformation. In general, a ^3^*J*_H1′−H2′_ value <2 Hz is suggestive of a C3′-*endo* sugar pucker (North type), whereas ^3^*J*_H1′−H2′_ value >8 Hz is characteristic of a C2′-*endo* pucker (South type) (61). The ^3^*J*_H1′−H2′_ values for **1a** and **1b** are summarized in Table 2 and the representative spectra are shown in Supplementary Figure S2. In the absence of cytosine methylation, all ribose rings in **1a** exhibit a C3′-*endo* conformation (^3^*J*_H1′−H2′_ ∼1–2 Hz), which is the preferred sugar puckering mode for A-form RNA duplex. Upon m^5^C methylation (**1b**), however, the 5′-terminal cytosine (C1) undergoes a sugar pucker switch from C3′-*endo* to C2′-*endo*, as evidenced by a large increase in ^3^*J*_H1′−H2′_ from 1.1 Hz to 8.2 Hz. This is accompanied with a switch in sugar puckering of the 3′-terminal guanosine (G6) to an intermediate conformation between C3′-*endo* and C2′-*endo* (^3^*J*_H1′−H2′_ = 6.0 Hz). No changes in sugar pucker mode are observed for the four internal residues G2 to C5, which continue to adopt a C3′-*endo* orientation. Our data support the findings of an earlier NMR study (45) and, together, they demonstrate that m^5^C can alter the sugar pucker conformation of the 5′- and 3′-terminal residues in CpG RNA duplexes.

To confirm our results, we further analysed the intensity of intranucleotide NOEs between the base protons (H6 in cytosine; H8 in guanine) and the sugar protons (H2′ and H3′), which correlates with the distance between the protons (Figures 2C, D and Table 2) (62,63). Consistent with our ^3^*J*_H1′−H2′_ data, the C1 residue of **1b** displays a strong NOE between its base proton H6 and sugar proton H2′ (∼2.2 Å) and a weak NOE between H6 and H3′ (∼3.9 Å), which is indicative of a C2′-*endo* conformation, and the G6 residue of **1b** shows approximately similar NOE intensities for H8-H2′ (∼2.7 Å) and H8-H3′ (∼3.3 Å), suggesting an intermediate C2′-*endo*/C3′-*endo* conformation. In comparison, both the C1 and G6 residues of unmethylated **1a** exhibit a C3′-*endo* sugar pucker, thus m^5^C methylation indeed produces a switch in the terminal sugar pucker orientation.

**Figure 3.**
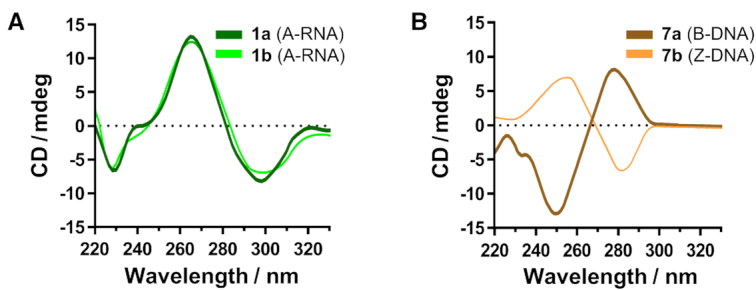
m^5^C methylation induces distinctly different conformational effects on RNA and DNA duplexes. Superimposition of the CD spectra of (**A**) CpG RNA duplex **1a** with that of its m^5^C-methylated counterpart **1b**, and (**B**) CpG DNA duplex **7a** with that of its m^5^C-methylated equivalent **7b**. Under physiologically-relevant salt and pH conditions, m^5^C methylation has negligible effect on the overall conformation of **1a**. In sharp contrast, methylation of **7a** triggered a transformation from right-handed B-DNA to left-handed Z-DNA, as apparent from the characteristic inversion of its CD spectrum.

Notably, however, these structural perturbations appear to have little or no effect on the overall secondary structure of the RNA duplex. This is apparent from CD analysis where the spectra of **1a** and **1b** exhibit characteristics which are typical of a right-handed A-form duplex, and they superimpose almost perfectly with each other (Figure 3A). Hence, m^5^C modification likely only affects the local nucleotide geometry without altering the overall conformation of CpG RNA duplexes.

### m^5^C-induced terminal sugar pucker switch is likely unique to alternating CpG RNA sequences

We next examined whether m^5^C could also elicit terminal sugar pucker switch in other RNA sequence contexts. To this end, we determined the ^3^*J*_H1′−H2′_ coupling constant values for the 5′- and 3′-terminal residues of each sequence (Table 1). Our results revealed a similar C2′-*endo* to C3′-*endo* switch in the longer 12-mer CpG duplex 2b, but not in the out-of-alternation CpG sequence **3a** and random sequences **4a–6a**. On the basis of these results, we performed further NMR experiments to investigate how the position of m^5^C methylation affects terminal sugar pucker conformation in alternating CpG duplexes **1a** and **2a**. The ^3^*J*_H1′−H2′_ values are summarised in Supplementary Table S4. For 6-mer CpG duplexes, terminal sugar switch occurred only when the third cytosine residue (C3) was methylated (i.e. **1b**), and not when the first (**1c**) or second (**1d**) cytosine residue was methylated. Similarly for 12-mer CpG duplexes, m^5^C-induced sugar switch was observed only when methylation was on C3 (**2b**), and not on C1 (**2c**) or C5 (**2d**), thus the structural effect of m^5^C is highly-dependent on the position of m^5^C methylation.

The sequence requirements and mechanisms by which m^5^C induces terminal sugar pucker switch are unclear at present and require further investigations. Nevertheless, the present study, the first to investigate the structural influence of m^5^C on multiple RNA sequence contexts, suggests that this conformational effect might be unique to alternating CpG RNA sequences.

### m^5^C triggers a B-to-Z DNA transformation in CpG DNA duplex

We next investigated the structural influence of m^5^C on CpG DNA duplex **7a**, i.e. the DNA equivalent of **1a** under the same physiologically-relevant conditions. A preliminary analysis of the ^3^*J*_H1′−H2′_ values and sugar-base proton distances of **7a** and **7b** revealed a switch in sugar puckering of all guanosine residues from C2′-*endo* to C3′-*endo* mode, whilst all cytosine residues preserve their C2′-*endo* orientation, which is highly suggestive of a B-to-Z structural transformation (Table 3; the representative 2D NOESY spectrum of **7b** is shown in Supplementary Figure S3). As further evidence, CD analysis showed a characteristic inversion of the CD spectrum upon m^5^C methylation, confirming that m^5^C does indeed triggers a major B-Z structural transformation in CpG DNA duplex **7a** (Figure 3B). This result is notable because it demonstrates that m^5^C produces different structural outcomes on RNA and DNA duplexes.

**Table 3.** ^1^H NMR chemical shifts δ_H_ (ppm) for CpG DNA duplex **7a** (top) and its m^5^C-methylated counterpart **7b** (bottom) observed at 25°C in D_2_O with WATERGATE suppression. The representative 2D NOESY spectrum is given in Supplementary Figure S3. For calculation of sugar-base proton distances, see Supplementary Data

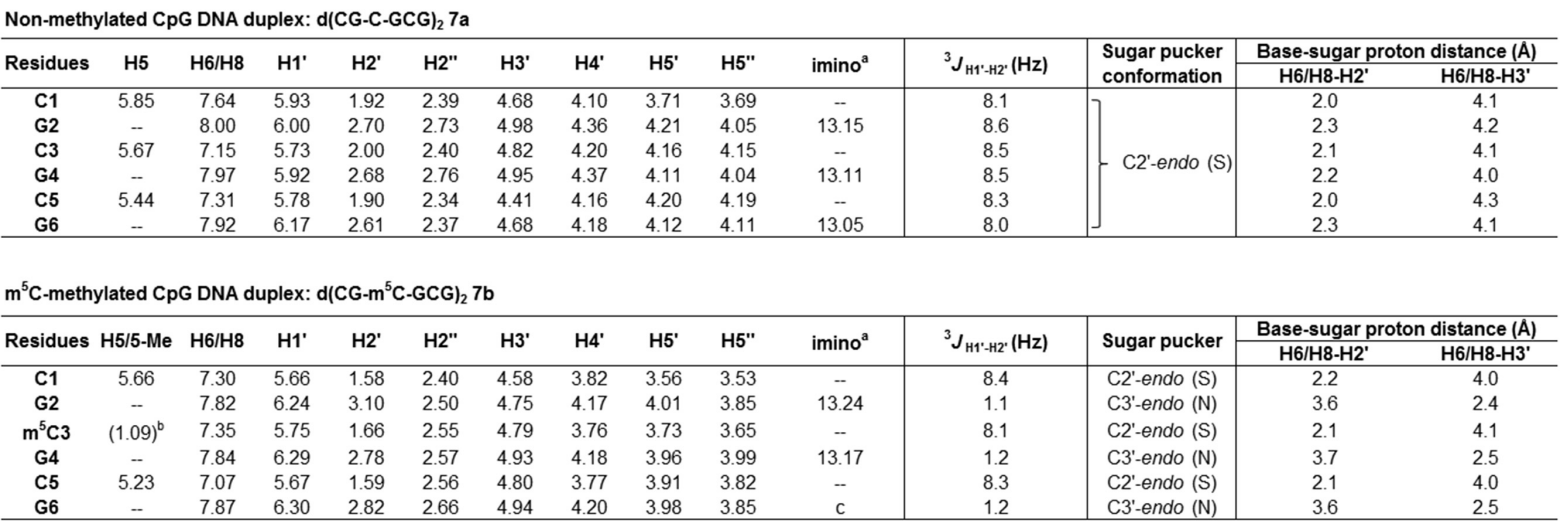

^a^Observed at 10°C in 9:1 buffer/D_2_O solvent mix. The buffer used was 10 mM sodium phosphate buffer (pH 7.4) containing 150 mM NaCl and 20 mM MgCl_2_.

^b^Chemical shift for 5-methyl group on m^5^C.

^c^Not observed even at 4°C.

The observed B-Z conversion is likely attributed to the ability of m^5^C to stabilize the Z-form DNA over its B-form structure (42,64). Indeed, UV-based thermodynamic analysis of **7a** showed that m^5^C methylation increases the stability of its Z-duplex by ∼2.5 kcal/mol relative to its B-duplex (compare Δ*G*°_310_ of **7a** with **7b**; Supplementary Table S3 and Supplementary Figure S4). Moreover, m^5^C-induced B-Z helicity change is primarily an entropy-driven process, as judged by the more favourable entropic contributions (ΔΔ*S*°_7a→7b_ = 31.6 cal/mol/K) which counteracts the enthalpy cost (ΔΔ*H*°_7a→7b_ = 7.3 kcal/mol). Further thermodynamic analysis of **7b** in the absence of MgCl_2_ (a known Z-conformation inducer) revealed a free energy of B-to-Z transition (ΔΔ*G*°_T_) of ∼1.5 kcal/mol (compare Δ*G*°_310_ of **7b** with 7b*); since **7b** does not undergo B-Z conversion in the absence of MgCl_2_, any free energy differences observed at these two salt conditions, i.e. between **7b** and 7b* is largely ascribed to conformational transition (Supplementary Table S3 and Supplementary Figure S5).

### m^5^C does not promote A–Z transition in CpG RNA duplex

To date, the effects of m^5^C methylation on the stability of Z-form RNA is unclear. We are also not aware of any prior studies investigating whether m^5^C could promote an analogous A–Z transition in RNA duplex. Therefore, we performed further CD experiments to monitor the conformation of CpG RNA duplex **1a** and its methylated equivalent **1b** at a range of MgCl_2_ concentrations (1–300 mM). The results showed that both **1a** and **1b** maintain an A-type double helical structure at all MgCl_2_ concentrations investigated (Supplementary Figure S6). In fact, the methylated RNA **1b** could not be induced to form Z-RNA even at MgCl_2_ concentration as high as 300 mM. Consistent with this observation, the presence of m^5^C does not significantly improve the stability of **1a** (ΔΔ*G*°_310_ ∼0.8 kcal/mol; Supplementary Table S3). Thus m^5^C methylation favours B–Z conversion in CpG DNA duplex, but not A–Z transition in CpG RNA duplex under the same physiological salt and pH conditions.

Overall, our results demonstrated that m^5^C methylation can directly influence the conformation of RNA and DNA duplexes. In the case of CpG RNA hexamer, m^5^C modification induces a local distortion of the phosphate backbone and a C3′-*endo* to C2′-*endo* terminal sugar pucker switch whereas, in CpG DNA hexamer, m^5^C triggers a transformation from a right-handed B-DNA to a left-handed Z-DNA. We appreciate that detailed crystallographic studies are required to fully characterise these structural changes, nevertheless, our available data clearly demonstrated that m^5^C modification produces distinctly different ‘structural signatures’ on RNA and DNA duplexes, even for duplexes with identical CpG sequence. The significance of this finding is unclear at present, however it might infer a possible involvement of m^5^C-induced conformational change in biological regulatory processes.

### Design principle of m^5^C-switchable probes

Inspired by these interesting findings, we envisaged that the different structure-remodelling effects of m^5^C on RNA and DNA duplexes could be exploited for the design of ‘methylation-switchable probe’ useful for studying m^5^C methylation in DNA and RNA. Notably, at present, no methods exist which permit the direct analysis of m^5^C MTase activity in living cells. There are also no reports of assay methods that are able to distinguish between the activities of DNA m^5^C MTases and RNA m^5^C MTases.

As a proof-of-principle study, we developed a novel m^5^C-probe which is able to fluoresce spontaneously in response to m^5^C-induced terminal sugar pucker switch, hence useful for sensing RNA:m^5^C MTase activity. The probe (**8a**; Figure 1) is modelled after the CpG RNA duplex **1a** but contains two important modifications. First, in order to visualise m^5^C-induced sugar pucker switch, the 5′-cytosine residues on the forward strand was replaced with the fluorescent cytosine analogue 6-phenylpyrrolocytosine (^p^C) (47,48). ^p^C is well-suited as reporter for our probe because it is highly fluorescence (quantum yield (*Φ*_F_) 0.31) and its emission is extremely sensitive to microenvironment changes (65–67). In particular, previous photophysical studies showed that ^p^C emits brightly when in a single-stranded environment, but becomes significantly quenched when hybridised with its complementary strand (65–67). Second, to render the m^5^C-probe sufficiently stable for live-cell applications, the RNA backbone was replaced with a 2′-*O-*methyl backbone, which is reasonably resistant to cellular nuclease degradation.

The analytical principle of the m^5^C-probe is illustrated in Figures 1A and B. By design, when the probe is unmethylated, the ^p^C fluorophore is able to base-pair with guanine and stack strongly with its adjacent base. This results in efficient quenching of ^p^C fluorescence through photoinduced electron transfer (PET). m^5^C methylation of the probe by RNA:m^5^C MTases, however, is expected to trigger a spontaneous C3′-*endo* to C2′-*endo* sugar pucker switch in ^p^C. Since the sugar ring pucker defines the glycosidic bond angle, a change in sugar puckering mode will also convert the orientation of ^P^C base from axial to equatorial, thereby disrupting its base-pairing and base-stacking interactions, leading to fluorescence activation.

### The m^5^C-probe has the sensitivity to detect a single m^5^C mark change

To verify our probe design, we first compared the fluorescence emission of the probes at different m^5^C methylation levels (λ_ex_ 360 nm; λ_em_ 465 nm; Supplementary Figure S7). Consistent with our detection strategy, the starting unmethylated probe **8a** displayed weak fluorescence, with a relatively low fluorescence quantum yield (*Φ*_F_) of ∼0.03, whilst its methylated counterpart **8b** yielded a 2-fold increase in fluorescence intensity (*Φ*_F_ ∼0.06; Supplemental Figure S7). Similarly the unmethylated probe **8c**, which was designed to simultaneously exploit sugar pucker switch at both 5′-ends, also gave negligible fluorescence response (*Φ*_F_ ∼0.04; Supplementary Figure S7). This result is notable because most DNA/RNA duplexes are known to undergo transient opening of the terminal base-pairs in solution. Conceivable, this might cause auto-activation of ^p^C fluorescence, which will severely limit the applications of these probes. Nevertheless, our fluorescence data from starting probes **8a** and **8c** suggests that emission arising from such transient end-fraying event is relatively insignificant. Indeed, with the improved probe **8c**, we are able to clearly detect a 2.8-fold increase in fluorescence intensity upon m^5^C methylation (**8d**; *Φ*_F_ ∼ 0.11; Supplemental Figure S7), which is remarkable in light of only a single m^5^C mark change. Thus our m^5^C-probe has the sensitivity to detect subtle methylation changes. Moreover, the introduction of a second m^5^C modification, i.e. **8e** led to approximately a doubling of fluorescence emission (5.3-fold), hence the fluorescence intensity of the probe increases proportionately with m^5^C methylation level.

**Figure 4.**
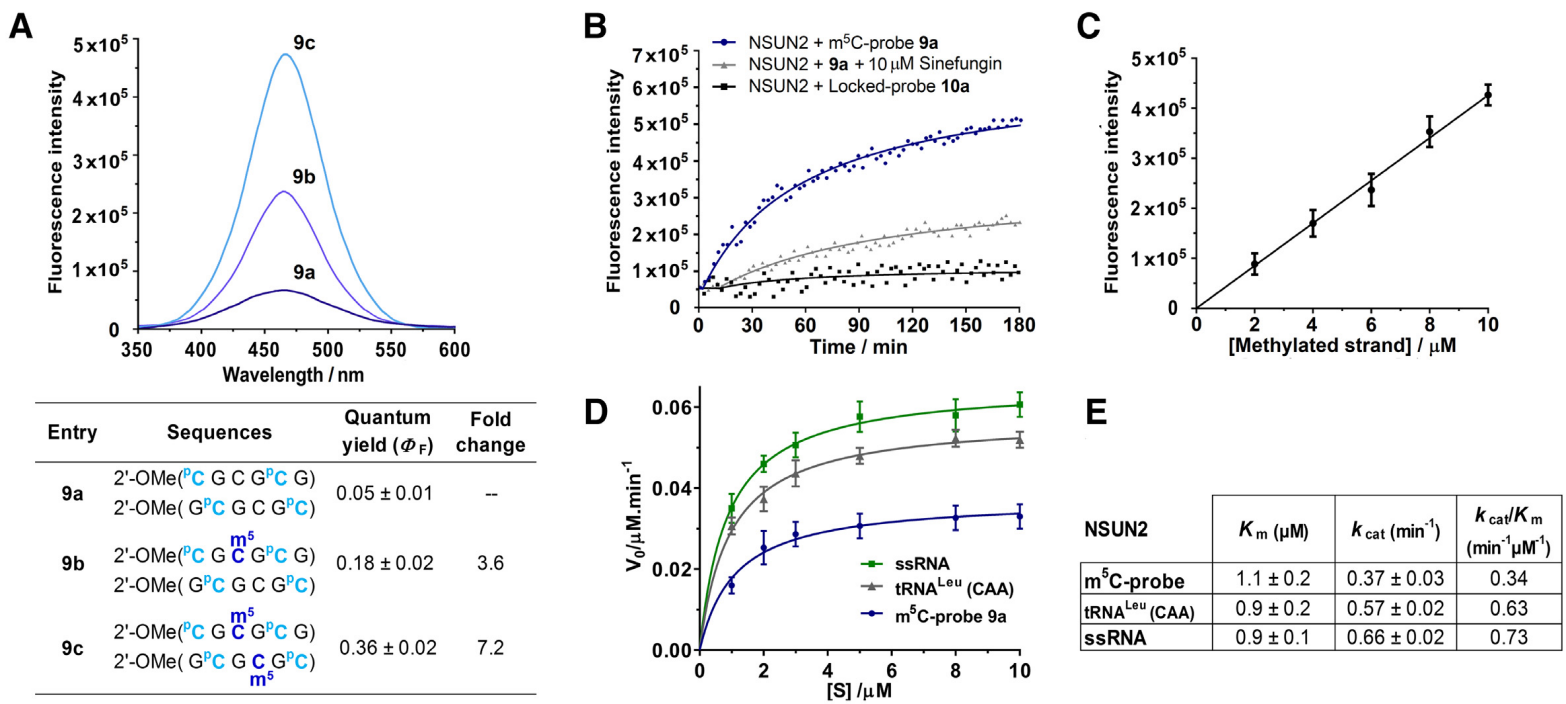
Verification of the m^5^C-probe design. (**A**) The fluorescence emission spectra of probe **9a** (λ_ex_ 360 nm; λ_em_ 465 nm) were recorded at 5 μM strand concentration in 10 mM sodium phosphate buffer (pH 7.4) containing 150 mM NaCl and 20 mM MgCl_2_, 37°C. The probe is highly responsive to its m^5^C methylation level; introduction of one and two m^5^C modifications led to a considerable 3.6-fold (**9b**) and 7.2-fold (**9c**) increase in fluorescence intensity, respectively. (**B**) Time-course fluorescence analysis of **9a** (5 μM). Significant fluorescence could be detected after a short 30-minute incubation with NSUN2 (0.5 μM; blue line); maximum emission was reached after ∼3 h, giving ∼8 fold increase in fluorescence intensity. Probe fluorescence reduces significantly in the presence of generic MTase inhibitor sinefungin (10 μM; grey line), thus the probe is specifically activated by NSUN2 MTase activity. Incubation of NSUN2 with locked-probe **10a** (5 μM; black line) gave negligible fluorescence response, confirming that probe activation is dependent on a change in sugar puckering of ^P^C. (**C**) The fluorescence intensity increases linearly with the concentration of methylated probe, this enables a direct read-out of NSUN2 MTase activity. Data are expressed as mean ± SD of three replicates. (**D**, **E**) Steady-state kinetics analyses of the methylation of m^5^C-probe, human tRNA^Leu^(CAA) (5′-CCAGACUCAAGUUCUGG-3′) and CpG-rich ssRNA (5′-CGCGCGCGCGCG-3′) by NSUN2. Data are expressed as mean ± SD of three replicates.

Interestingly, the fluorescence output of the probe could be further increased by incorporating additional ^p^C fluorophores, as demonstrated by probe **9a** (Figure 4A) where the concurrent replacement of cytosine 1 and 5 in both strands with ^p^C led to significant improvements in fluorescence light-up response (Δ*Φ*_F_ (**9b**) 3.6-fold; (9c) 7.2-fold). Despite the introduction of 2′-*O*-Me backbone and four relatively bulky ^p^C residues, NMR data indicate that both the 5′- and 3′-terminal residues of probe **9a** continue to adopt a C3′-*endo* pucker conformation (^3^*J*_H1′−H2′_ ∼1–2 Hz; Supplementary Table S4), and they spontaneously assume a C2′-*endo* pucker orientation (^3^*J*_H1′−H2′_ ∼8 Hz) upon m^5^C methylation (9c), thus these modifications do not interfere with fluorescence activation of the probe. There is also minimal disturbance to the overall duplex structure of probe **9a** relative to parent probes **8a** and **1a**, as verified by CD analysis (Supplementary Figure S8). Amongst the probes investigated, **9a** gives the greatest fluorescence response, it was therefore selected as representative m^5^C-probe for subsequent evaluations.

### The m^5^C-probe is highly-selective for NSUN2 over other RNA/DNA:m^5^C MTases

We next examined whether m^5^C-probe **9a** could function as fluorogenic substrate for RNA:m^5^C MTase by testing it against NSUN2, which is of particular interest in light of emerging evidence linking NSUN2 with m^5^C methylation of human mRNA (14) and its association with human cancers (25–29). In a typical assay, 5 μM of **9a** was incubated with NSUN2 (0.5 μM) and methyl donor *S-*adenosyl-l-methionine (SAM; 200 μM) under physiologically-relevant conditions (at 37°C in 50 mM HEPES buffer containing 150 mM NaCl and 20 mM MgCl_2_, pH 7.4). The formation of methylated probe was measured by an increase in fluorescence signal at 465 nm (λ_ex_ 360 nm). Our result showed that the probe could be readily methylated by NSUN2, yielding ∼8-fold increase in fluorescence signal after 3 h (Figure 4B).

To rule out fluorescence activation due to other non-specific mechanisms, we repeated the assay in the absence of NSUN2 or methyl donor SAM; in both cases, there was no detectable fluorescence light-up response (Supplementary Figure S9). Furthermore, pre-incubation of NSUN2 with 10 μM of sinefungin (a non-specific DNA/RNA MTase inhibitor) (25,68,69) resulted in a significant reduction in fluorescence intensity, confirming that fluorescence activation was specifically mediated by NSUN2 MTase activity (Figure 4B). Notably, the fluorescence intensity increases linearly with the concentration of methylated probe, thus providing a direct read-out of NSUN2 MTase activity (Figure 4C).

We next verified whether m^5^C-induced sugar pucker switch in ^P^C was indeed responsible for fluorescence turn-on. For this purpose, we synthesised ‘locked-probe’ **10a** (Figure 1C) wherein the ^p^C fluorophore was conformationally restricted in a C3′-*endo* sugar pucker orientation *via* a methylene bridge connecting O2′ with C4′ (^L^C; Figure 1C). As a result of this modification, the locked-probe will no longer be able to undergo sugar pucker switch upon m^5^C methylation. Consistent with our hypothesis, **10a** gave negligible fluorescence light-up response when exposed to NSUN2 (Figure 4B), even though MALDI-TOF MS assay clearly showed that this probe can be methylated by NSUN2 (Supplementary Figure S10). Thus probe activation mechanism is strictly dependent on a change in sugar pucker mode of the 5′-^p^C residues.

We then evaluated the specificity of probe **9a** by testing it against a panel of human RNA:m^5^C MTases, including three structurally-related homologues NSUN3, NSUN5A, and NSUN6, as well as DNMT homologue TRDMT1 (formerly DNMT2) (6,22). In all cases, there was no significant fluorescence activation (Supplementary Figure S9). Furthermore, MALDI-TOF MS assay showed no methylated product formation even after a prolonged 8 h-incubation with these enzymes, suggesting that **9a** is highly selective for NSUN2 over other RNA:m^5^C MTases (Supplementary Figure S10). Remarkably, **9a** also gave no appreciable fluorescence response and methylated product when exposed to key human DNA:m^5^C MTases, namely DNMT1 and DNMT3A (21,22), hence the probe is also able to discriminate against DNA:m^5^C MTases (Supplementary Figures S9 and S10). Steady-state kinetic analysis using MALDI-TOF MS assay indicated that the m^5^C-probe is a relatively good substrate for NSUN2 (*k*_cat_*/K*_m_ = 0.3 min^-1^μM^-1^), and is only ∼2-fold less efficiently methylated compared with its native tRNA substrate, tRNA^Leu^(CAA), and CpG-rich ssRNA (Figures 4D and E).

### The m^5^C-probe is applicable for live-cell imaging, single-cell flow cytometry analysis and cell-based inhibitor screening

**Figure 5.**
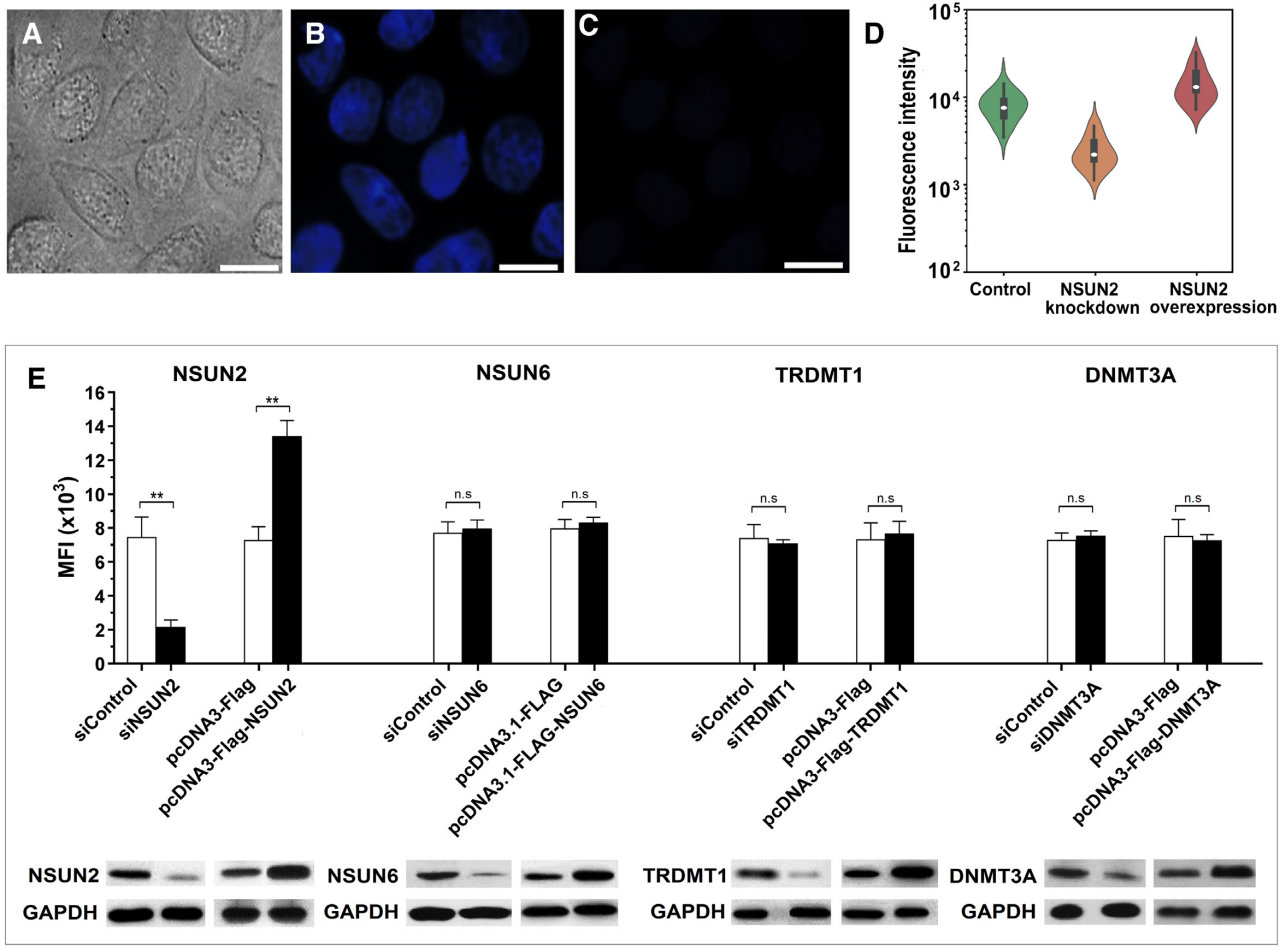
The m^5^C-probe provides real-time visualisation of cellular NSUN2 MTase activity. Live cell imaging of HeLa cells following treatment with m^5^C-probe **9a** (10 μM) using (**A**) bright field and (**B**) fluorescence microscopy. (**C**) Fluorescence image of HeLa cells treated with locked-probe **10a** (10 μM) obtained from a separate experiment than the correlated images in panels A and B. It is displayed using the same brightness and contrast settings as that in panel B. Scale bar, 20 μm. (**D**) Violin plots showing the flow cytometry analysis of HeLa cells transfected with probe **9a** (green), and with NSUN2 knockdown (orange) or NSUN2 overexpression (red) (λ_ex_ 355 nm; λ_em_ 425–475 nm). The boxes in violin plots show the median (white dot), 25–75 percentile (black box) and 5–95 percentile values (black bar). (**E**) The fluorescence intensity of m^5^C-probe changes with the expression levels of NSUN2 in HeLa cells, but is unresponsive towards NSUN6, TRDMT1 and DNMT3A. A minimum of 20 000 live cells were analysed for determination of mean fluorescence intensity (MFI). Data are expressed as mean ± SD of three biological replicates. **P < 0.01; n.s. = not significant.

On the basis of these promising data, we proceeded to investigate whether the m^5^C-probe strategy could provide direct visualisation of NSUN2 activity in living cells. Accordingly, HeLa cells, which constitutively express NSUN2, were transfected with either **9a** (10 μM) or the locked-probe **10a** (control) *via* Lipofectamine 2000, and then imaged using fluorescence microscopy (λ_ex_ 340–380 nm, λ_em_ 435–485 nm). Cells treated with probe **9a** gave off bright blue fluorescence after 1 h; this is ∼7-fold higher intensity compared with control (Figures 5A–C).

Further characterisation of the probe by time course fluorescence analysis showed that the probe remained strongly emissive in HeLa cell lysate for at least 3 h, suggesting good photostability (Supplementary Figure S11A). Moreover, there was no obvious increase in probe fluorescence in cell lysate which had been spiked with 1 mM sinefungin, suggesting no significant fluorescence arising from NSUN2-independent mechanisms (Supplementary Figure S11A). The lack of fluorescence increase further suggests that the probe is reasonably resistant to cellular degradation, which would otherwise release unstacked fluorophore. Consistent with this result, a repeat of the cell lysate experiment using locked-probe **10a** gave very little or no increase in fluorescence in HeLa cell lysate, both in the presence and absence of 1 mM sinefungin (Supplementary Figure S11B). These data suggest that probe degradation by cellular nucleases and other nucleic acid modifying enzymes do not interfere with the performance of the m^5^C-probe **9a** significantly, at least not within the time frame of our cell-based assay (typically 1 h). Furthermore, the probe is relatively non-cytotoxic, as evidenced by MTT toxicity assay and cell morphological assessment, where >80% of the cells remained viable after being exposed to 40 μM probe for 24 h (Supplementary Figure S11C).

Besides live-cell imaging, the m^5^C-probe is also suitable for use in single-cell flow cytometry analysis (λ_ex_ 355 nm; λ_em_ 425–475 nm). In particular, NSUN2 knockdown in HeLa cells caused a marked reduction in probe fluorescence, whereas overexpression of NSUN2 led to significant increase in fluorescence signal (Figures 5D and E). Remarkably, the fluorescence intensity was unchanged with NSUN6, TRDMT1, and DNMT3A knockdown and overexpression, thus, consistent with our *in vitro* data, the probe is highly selective for NSUN2 over other m^5^C MTases. Prior to this work, we are not aware of any assay method that is able to discriminate between DNA and RNA m^5^C MTase activities in cells.

**Figure 6.**
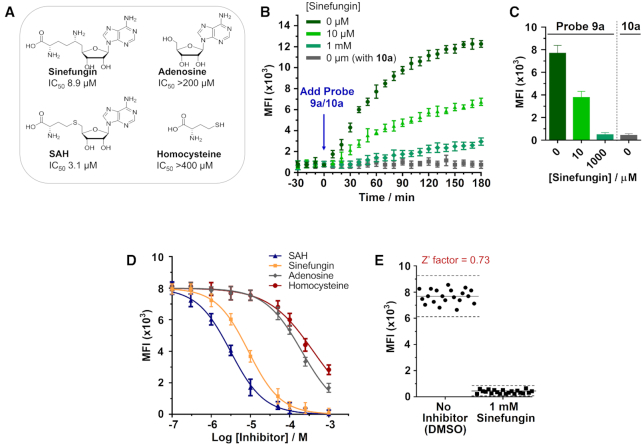
Application of the m^5^C-probe assay in cell-based screening of NSUN2 inhibitors. (**A**) IC_50_ values of non-specific MTase inhibitors against NSUN2. Results are from m^5^C-probe assay. (**B**) Analysis of NSUN2 inhibition in live HeLa cells by real-time flow cytometry (λ_ex_ 355 nm; λ_em_ 425–475 nm). Cells were pre-incubated with sinefungin (0, 10 and 1000 μM; colour coded) for 30 min prior to treatment with m^5^C-probe **9a** (10 μM) or locked-probe **10a** (10 μM; control). (**C**) The flow cytometry profile at 1 h showed a concentration-dependent decrease in mean fluorescence intensity (MFI) in cells treated with **9a**. (**D**) Inhibition of NSUN2 by selected MTase inhibitors. The m^5^C-probe assay could distinguish known inhibitors from structurally-related negative controls. Data are expressed as mean ± SD of three replicates. (**E**) Screening validation of the m^5^C-probe assay in a 96-well format. The assay has a *Z*’ factor of 0.73, which demonstrates excellent reproducibility.

Finally, the developed m^5^C-probe could also be used to directly measure NSUN2 inhibition in cells. Treatment of HeLa cells with increasing concentrations of known MTase inhibitors, sinefungin or *S*-adenosyl-L-homocysteine (SAH), for 30 min prior to the addition of probe **9a** (10 μM) led to a concentration-dependent decrease in fluorescence signal (Figures 6A–C). The determined IC_50_ values of sinefungin (8.9 μM) and SAH (3.1 μM) against NSUN2 are comparable with their inhibitory activities against other RNA MTases (IC_50_ values typically range between 0.1 μM to 20 μM (25,68); Figure 6D). Importantly, the negative controls, adenosine and homocysteine, showed poor inhibition towards NSUN2, as anticipated (IC_50_s >200 μM and >400 μM, respectively). Thus our assay is able to identify known NSUN2 inhibitors from amongst structurally-related analogues. We further performed Z’ factor analysis (55) to evaluate the reliability of our assay for high-throughput screening of inhibitor (for experimental details, see Supplementary Data). Our assay produces an average *Z*’ factor of 0.73 in a 96-well plate format, indicating that it has the required statistical reproducibility for cell-based screening of NSUN2 inhibitors (Figure 6E).

### The m^5^C-switchable probe strategy could be adapted for the study of DNA:m^5^C MTase activity

**Figure 7.**
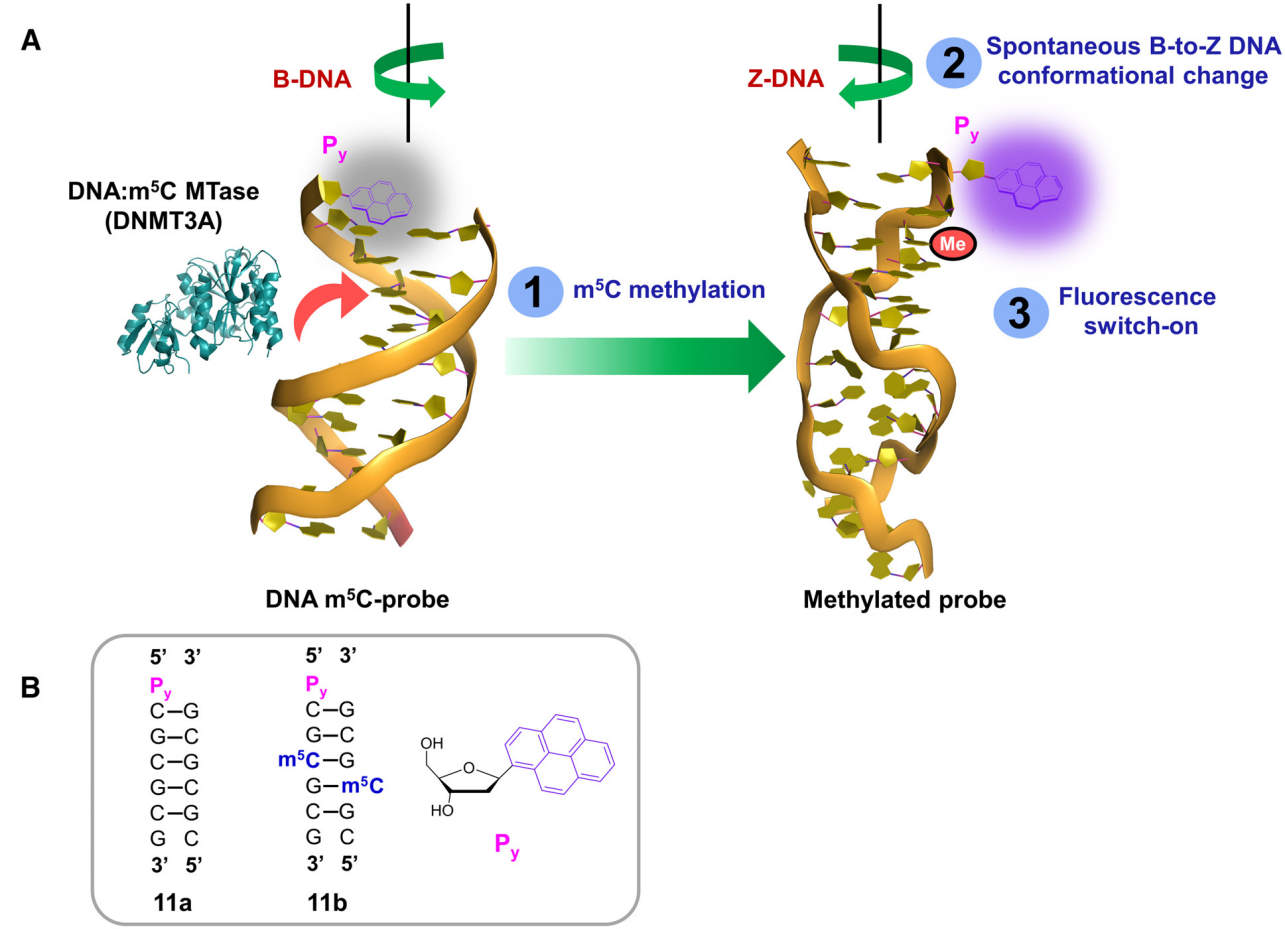
The m^5^C-probe approach could be adapted for the detection of DNA:m^5^C MTases activity. (**A**) The DNA m^5^C-probe **11a** is conformationally-responsive to m^5^C methylation. It exists as a right handed B-DNA structure when unmethylated, but undergoes spontaneous and rapid transformation to the more compact, left-handed Z-DNA structure upon m^5^C methylation by DNA:m^5^C MTases (e.g. DNMT3A). Such a major B-Z conversion severely disrupts end-stacking interaction of the fluorophore, pyrene deoxyriboside (P_y_), leading to fluorescence activation. (**B**) Schematic representation of DNA m^5^C-probe **11a** and its methylated counterpart **11b**. The structure of P_y_ monomer is shown.

In light of present finding that m^5^C methylation can induce a spontaneous B–Z conformational change in CpG DNA duplex **7a** (Figure 3B and Supplementary Figure S6), our m^5^C-probe strategy may, in principle, also be adapted for the study of DNA:m^5^C MTase activity. To examine this possibility, we prepared DNA probe **11a**, which is an analogue of **7a** containing a 5′-overhang pyrene deoxyriboside (P_y_) in the forward strand (Figure 7). The design of this probe exploits differences in the ability of P_y_ to participate in end-stacking interactions in the B-DNA and Z-DNA forms. It is clear, from a number of studies, that whereas the B-DNA (diameter of helix ∼20 Å) is able to adopt a continuous base-stacking arrangement, the more compact Z-DNA (diameter of helix ∼18 Å) only permits discontinuous series of four-base stack (70). Because of these inherent differences in base-stacking pattern, the unpaired P_y_ will only be able to end-stack on adjacent G:C base pair when it is in a B-DNA environment, and not in the Z-DNA environment. Accordingly, conversion of probe **11a** from B-DNA to Z-DNA form upon m^5^C methylation is expected to cause a destacking of P_y_ fluorophores and, consequently, fluorescence activation.

**Figure 8.**
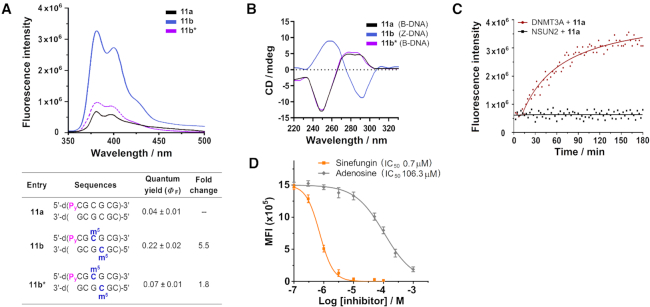
DNA probe **11a** provides highly sensitive detection of DNA:m^5^C methylation. (**A**) Fluorescence measurement of probe **11a** (5 μM) showed two distinct pyrene emission peaks at 380 nm and 400 nm (λ_ex_ 340 nm). The spectra were recorded at 37°C under physiologically-relevant conditions (10 mM sodium phosphate buffer (pH 7.4) containing 150 mM NaCl and 20 mM MgCl_2_). The probe exhibited a significant 5.5-fold increase in fluorescence intensity upon m^5^C methylation (**11b**), but gave no appreciable light-up response in the absence of MgCl_2_ (11b*), implying that fluorescence switch-on was primarily mediated by B–Z structural conversion of the probe. (**B**) m^5^C methylation triggered a major inversion of the CD spectrum of probe **11a** under physiologically-relevant conditions. There was little or no conformational change in the absence of MgCl_2_ (11b*). (**C**) Time-course fluorescence analysis (λ_ex_ 340 nm; λ_em_ 400 nm) of probe **11a** (5 μM) in the presence of DNMT3A (0.5 μM; red line) or NSUN2 (0.5 μM; black line). The results demonstrate that probe **11a** is selectively activated by DNMT3A, and not by NSUN2. (**D**) Probe **11a** could be used for *in vitro* inhibition assay of DNMT3A. Both sinefungin (known DNMT3A inhibitor) and adenosine (negative control) displayed clear concentration-dependent decrease in fluorescence, with determined IC_50_ values of 0.7 μM and 106.3 μM, respectively. Data are expressed as mean ± SD of three replicates.

Consistent with our probe detection strategy, m^5^C methylation readily triggered a B-Z transformation in probe **11a**, with concomitant increase in fluorescence emission (Δ*Φ*_F_ ∼5.5-fold; λ_ex_ 340 nm; λ_em_ 380 nm and 400 nm; Figures 8A and B). Notably, there was no fluorescence activation in the absence of MgCl_2_ where 11a* did not undergo B-to-Z conversion, thus the observed fluorescence response was primarily mediated by a B–Z conversion of the probe (Figure 8B). Moreover, our thermodynamic analysis supports the notion that B-Z transition triggers a destacking of the dangling P_y_ (Supplementary Table S3). In particular, in the absence of m^5^C methylation, P_y_ is able to stack readily at the 5′-end of probe **11a**, giving an end-stacking stabilisation of ∼2.8 kcal/mol (compare the difference in duplex stability between **11a** and **7a**; Supplementary Table S3 and Supplementary Figures S12 and S13). Upon m^5^C methylation, however, end-stacking interaction is completely abolished (negligible difference in stability between **11b** and **7b**). This suggests that the end-stacking property of P_y_ and, hence, its fluorescence output is sensitive to m^5^C methylation status.

Preliminary biochemical analysis further demonstrated that probe fluorescence is only activated by DNA:m^5^C MTases DNMT3A, and not by RNA:m^5^C MTases NSUN2 (Figure 8C). Importantly, m^5^C-probe **11a** may also be applied to the inhibition study of DNMT3A. As shown in Figure 8D, incubation of probe **11a** (5 μM) and DNMT3A (0.5 μM) with increasing concentrations of sinefungin (known DNMT3A inhibitor) or adenosine (negative control) for 30 min led to clear concentration-dependent decrease in fluorescence intensity. The determined IC_50_ values of sinefungin (0.7 μM) and adenosine (106.3 μM) are comparable with previously reported values (71), thus the developed m^5^C-probe strategy could be used for *in vitro* screening of DNMT3A inhibitors. Work is currently underway to explore the possibility of employing both probes concurrently for the simultaneous detection of RNA and DNA m^5^C MTase activity.

## CONCLUSION

Overall, we use a combination of NMR, CD and thermodynamic analyses to investigate the structural impact of m^5^C methylation on RNA and DNA duplexes. Our results revealed that, although m^5^C does not hinder canonical Watson–Crick base-pairing interactions, this modification can, in fact, elicits significant conformational change in certain RNA and DNA sequence contexts. We further demonstrated that m^5^C produces distinctly different ‘structural signatures’ on RNA and DNA duplexes, including duplexes with identical CpG sequences. In the case of CpG RNA duplex (as exemplified by **1a**), m^5^C methylation induces a local distortion of the phosphate backbone and a C3′-*endo* to C2′-*endo* sugar pucker switch in the terminal residues. However, in the case of CpG DNA duplex (as exemplified by **7a**), m^5^C triggers a remarkable B-Z structural transformation. The significance of this result is unclear at present nevertheless, given that majority of the m^5^C modifications in DNA and mRNA are distributed within the CpG consensus motif, sequence information alone is insufficient to discriminate m^5^C marks on RNA and DNA CpG sites. Therefore, one question which arises from this finding is whether the duplex-remodelling property of m^5^C might provide a mechanism for its specific recognition by RNA/DNA:m^5^C binding proteins.

On the basis of this interesting finding, we further provided proof-of-principle that m^5^C-induced conformational change can be exploited for direct detection of m^5^C MTase activity in cells. This was demonstrated by the development of the first m^5^C-responsive probe **9a**, which switches sugar pucker conformation spontaneously according to its m^5^C methylation status, hence useful for sensing RNA:m^5^C MTase activity.

The m^5^C-probe is simple, inexpensive and highly selective for NSUN2 over other RNA:m^5^C MTases (including NSUN3, NSUN5A, NSUN6 and TRDMT1) and DNA:m^5^C MTases (including DNMT1 and DNMT3A). Through the use of this probe, we achieved fluorescence imaging and real-time flow cytometry analysis of NSUN2 activity in live HeLa cells. We further demonstrated the utility of the probe in cell-based screening of NSUN2 inhibitors. Prior to this study, we are not aware of any methods that permit direct sensing of RNA:m^5^C MTase activity in cells. There are also no reports of assays that selectively target NSUN2 over other RNA/DNA:m^5^C MTases. The discovery of such highly selective probes is rarely achieved and may prove valuable in advancing our knowledge of NSUN2 in m^5^C-regulated processes. Importantly, our m^5^C-probe approach could also be adapted for the analysis of DNA:m^5^C MTase activity. This was demonstrated by the development of DNA m^5^C-probe **11a**, which is useful for *in vitro* screening of DNMT3A inhibitors.

We appreciate that m^5^C-induced terminal sugar pucker switch is likely an interesting anomaly that is unique to alternating CpG RNA duplexes, since this phenomenon was not observed in majority of other sequences investigated in this study. Therefore, one limitation of the proposed approach is that it is not applicable to RNA:m^5^C methyltransferases that do not methylate CpG substrates. Nevertheless, given the importance of NSUN2, both as a key regulator of m^5^C marks in mRNA and a potential therapeutic target, we envisaged that the proposed NSUN2 assay strategy would be of broad scientific interest.

## Supplementary Material

gkz1047_Supplemental_File
