# Supplementary material for: A general strategy exploiting m5C duplex-remodelling effect for selective detection of RNA and DNA m5C methyltransferase activity in cells
Source: Nucleic Acids Res. 2019 Nov 6;48(1):e5. doi: 10.1093/nar/gkz1047 (PMC7145549; doi:10.1093/nar/gkz1047)
Supplement: gkz1047_Supplemental_File [file gkz1047_supplemental_file.pdf]

Supplementary Data

A general strategy exploiting m^{5}C duplex-remodelling effect for selective detection of RNA and DNA m^{5}C methyltransferase activity in cells.

Tianming Yang[1], Joanne J. A. Low[1] and Esther C. Y. Woon[1,*]

[1] Department of Pharmacy, National University of Singapore, 18 Science Drive 4, 117543, Singapore.

* To whom correspondence should be addressed.

Esther C. Y. Woon

Email: esther.woon@nus.edu.sg; Tel: (+65) 6516 2932; Fax: (+65) 6779 1554.

Contents:

**Table S1.** Sequences of RNA and DNA duplexes investigated in this study and their MALDI-TOF MS data.	S4
**Table S2.** Sequences of fluorescent m^5C-probes investigated in this study and their MALDI-TOF MS data.	S5
**Table S3.** Melting temperature and thermodynamic parameters of selected RNA and DNA duplexes.	S6
**Table S4.** Effects of m^5C modification position on terminal sugar pucker conformation.	S7
**Figure S1.** Representative ^1H imino NMR spectra of methylated RNA duplex **1b** acquired at 4 °C and 10 °C.	S8
**Figure S2.** Representative 1D ^1H NMR spectra showing the homonuclear $^3J_{H1'-H2'}$ coupling constant for CpG RNA duplex **1a** and its m^5C-methylated counterpart **1b**.	S9
**Figure S3.** Representative 2D NOESY spectrum of the H2'/H3'-aromatic region of CpG DNA duplex **7b**.	S10
**Figure S4.** UV-based melting analysis of CpG DNA duplex **7a** and its m^5C-methylated counterpart **7b**.	S11
**Figure S5.** UV-based melting analysis of CpG DNA duplex **7a*** and its m^5C-methylated counterpart **7b*** *in the absence of Z-conformation inducer* $MgCl_2$.	S12
**Figure S6.** CD conformational analyses of CpG RNA and DNA duplexes at a range of $MgCl_2$ concentrations.	S13
**Figure S7.** Effects of m^5C methylation levels on fluorescence light-up response and fluorescence quantum yield of RNA m^5C-probe **8a**.	S14
**Figure S8.** Superimposition of the CD spectrum of m^5C-probe **9a** with that of its parent probes **8a** and **1a**.	S15
**Figure S9.** Time-course fluorescence intensity spectra demonstrated that fluorescence activation of m^5C-probe **9a** was specifically triggered by NSUN2.	S16
**Figure S10.** MALDI-TOF MS assays of the m^5C-probe **9a** against a panel of human RNA/DNA:m^5C MTases.	S17
**Figure S11.** The m^5C-probe **9a** demonstrates good cellular stability and low cytotoxicity.	S18
**Figure S12.** UV-based melting analysis of DNA m^5C-probe **11a** and its methylated counterpart **11b**.	S19
**Figure S13.** UV-based melting analysis of DNA m^5C-probe **11a*** and its methylated counterpart **11b*** *in the absence of Z-conformation inducer* $MgCl_2$.	S20

Materials and methods

Preparation of 2’-*O*-methyl RNA and DNA m^5C-probes S21

DNA/RNA methyltransferase inhibitors S21

Human m^5C:RNA MTases (NSUN2, NSUN3, NSUN5A and NSUN6 proteins) S21

Human m^5C:DNA MTases (DNMT1 and DNMT3A proteins) S22

Circular dichroism (CD) spectroscopy S22

UV-based thermal denaturation experiments S22

Thermodynamic parameters derived from UV-melting experiments S23

Determination of fluorescence quantum yields of m^5C-probes S23

MALDI-TOF MS-based methyltransferase assay S24

HeLa cell culture S25

siRNA gene silencing S25

Gene overexpression S25

Western blot analysis S26

Cell-based m^5C-probe methyltransferase assay S27

Determination of IC_{50} S27

Determination of Z’ factor S27

References S28

Table S1. Sequences of RNA and DNA duplexes investigated in this study and their MALDI-TOF MS data.

Entry		Sequences	MALDI-MS	
			$[M+H]^+$ (calculated)	$[M+H]^+$ (observed)
RNA	1a	5'-r(CG C G CG)-3'<br>3'-r(GC G C GC)-5'	3779.8	3779.2
	1b	m^5<br>5'-r(CG **C** G CG)-3'<br>3'-r(GC G **C** GC)-5'<br>m^5	3807.3	3807.0
	2a	5'-r(CG C GCGCGC G CG)-3'<br>3'-r(GC G CGCGCG C GC)-5'	7681.5	7681.4
	2b	m^5<br>5'-r(CG **C** GCGCGC G CG)-3'<br>3'-r(GC G CGCGCG **C** GC)-5'<br>m^5	7709.7	7709.1
	3a	5'-r(AU C G AC)-3'<br>3'-r(UA G C UG)-5'	3734.6	3734.5
	3b	m^5<br>5'-r(AU **C** G AC)-3'<br>3'-r(UA G **C** UG)-5'<br>m^5	3762.8	3762.3
	4a	5'-r(UC C G AU)-3'<br>3'-r(AG G C UA)-5'	3734.9	3734.1
	4b	m^5<br>5'-r(UC **C** G AU)-3'<br>3'-r(AG G **C** UA)-5'<br>m^5	3762.2	3762.8
	5a	5'-r(GU C G GA)-3'<br>3'-r(CA G C CU)-5'	3749.3	3749.5
	5b	m^5<br>5'-r(GU **C** G GA)-3'<br>3'-r(CA G **C** CU)-5'<br>m^5	3777.4	3777.9
	6a	5'-r(GC C UGACUA G UA)-3'<br>3'-r(CG G ACUGAU C AU)-5'	7591.1	7591.5
	6b	m^5<br>5'-r(GC **C** UGACUA G UA)-3'<br>3'-r(CG G ACUGAU **C** AU)-5'<br>m^5	7619.0	7619.7
DNA	7a	5'-d(CG C G CG)-3'<br>3'-d(GC G C GC)-5'	3587.5	3587.6
	7b	m^5<br>5'-d(CG **C** G CG)-3'<br>3'-d(GC G **C** GC)-5'<br>m^5	3615.6	3615.1

Table S2. Sequences of fluorescence m^{5}C-probes investigated in this study and their MALDI-TOF MS data. The fluorescent reporters used for the 2'-OMe RNA probe and DNA probe are 2'-*O*-methyl 6-phenylpyrrolocytidine (^{P}C) and pyrene deoxynucleoside (P_y), respectively. In the locked-probe, the ^{P}C fluorophore is conformationally restricted in a C3'-*endo* sugar pucker orientation *via* a O2'-C4' methylene linkage to give 'locked 2'-*O*-methyl 6-phenylpyrrolocytidine' (^{L}C).

Entry		Sequences	MALDI-MS	
			[M+H]$^+$ (calculated)	[M+H]$^+$ (observed)
2'-OMe RNA m^{5}C-probe	**8a**	2'-OMe(^{P}CG C G C G)<br>2'-OMe(GC G C G C)	4147.8	4147.0
	**8b**	m^5<br>2'-OMe(^{P}CG C G C G)<br>2'-OMe(GC G C G C)	4161.4	4161.3
	**8c**	2'-OMe(^{P}CG C G C G)<br>2'-OMe(GC G C G^{P}C)	4147.8	4147.1
	**8d**	m^5<br>2'-OMe(^{P}CG C G C G)<br>2'-OMe(GC G C G^{P}C)	4161.4	4161.2
	**8e**	m^5<br>2'-OMe(^{P}CG C G C G)<br>2'-OMe(GC G C G^{P}C)<br>m^5	4175.3	4175.7
	**9a**	2'-OMe(^{P}C G C G^{P}C G)<br>2'-OMe(G^{P}C G C G^{P}C)	4347.0	4347.2
	**9b**	m^5<br>2'-OMe(^{P}C G C G^{P}C G)<br>2'-OMe(G^{P}C G C G^{P}C)	4361.1	4361.6
	**9c**	m^5<br>2'-OMe(^{P}C G C G^{P}C G)<br>2'-OMe(G^{P}C G C G^{P}C)<br>m^5	4375.5	4375.0
Locked-probe	**10a**	2'-OMe(^{L}C G C G C G)<br>2'-OMe(G CG C G^{L}C)	4145.7	4145.8
	**10b**	m^5<br>2'-OMe(^{L}C G C G C G)<br>2'-OMe(G CG C G^{L}C)	4159.2	4159.5
	**10c**	m^5<br>2'-OMe(^{L}C G C G C G)<br>2'-OMe(G CG C G^{L}C)<br>m^5	4173.6	4173.3
DNA m^{5}C-probe	**11a**	5'-d(P_yCG CG C G)-3'<br>3'-d(G CG C G C)-5'	3967.9	3967.2
	**11b**	m^5<br>5'-d(P_yCG CG C G)-3'<br>3'-d(G CG C G C)-5'<br>m^5	3995.7	3995.4

Table S3. Melting temperature (T_m) and thermodynamic parameters of selected RNA and DNA duplexes. Under the same physiological salt and pH conditions, m^5C methylation triggered a dramatic transformation from a right-handed B-DNA to a left-handed Z-DNA in duplex **7a**, but not an analogous A-Z transition in duplex **1a**, even though both duplexes have identical CpG sequence. The DNA m^5C-probe **11a** undergoes similar B-Z conversion upon m^5C modification.

Entry	Sequences	Conformations	T_m (°C) (at 5 μM)[a]	Δ*H*° (kcal/mol)[b]	Δ*S*° (cal/mol/K)[b]	Δ*G*°$_{310}$ (kcal/mol)	ΔΔ*G*°$_{310}$ (kcal/mol)
1a	5'-r(CG C G CG)-3'<br>3'-r(GC G C GC)-5'	A-RNA	49.7 ± 0.5	-63.5 ± 1.1	-174.1 ± 2.2	-9.5 ± 0.1	-0.8
1b	m^5<br>5'-r(CG **C** G CG)-3'<br>3'-r(GC G **C** GC)-5'<br>m^5	A-RNA	51.9 ± 0.5	-60.4 ± 0.9	-161.5 ± 1.9	-10.3 ± 0.2	
7a	5'-d(CG C G CG)-3'<br>3'-d(GC G C GC)-5'	B-DNA	43.8 ± 0.2	-59.5 ± 2.1	-165.7 ± 2.5	-8.1 ± 0.1	-2.5
7b	m^5<br>5'-d(CG **C** G CG)-3'<br>3'-d(GC G **C** GC)-5'<br>m^5	Z-DNA	53.4 ± 0.2	-52.2 ± 1.7	-134.1 ± 4.6	-10.6 ± 0.2	
7a*	5'-d(CG C G CG)-3'<br>3'-d(GC G C GC)-5'	B-DNA	42.3 ± 0.3	-58.9 ± 1.3	-163.8 ± 2.5	-8.1 ± 0.2	-1.0
7b*	m^5<br>5'-d(CG **C** G CG)-3'<br>3'-d(GC G **C** GC)-5'<br>m^5	B-DNA	45.7 ± 0.3	-56.4 ± 1.5	-152.5 ± 4.6	-9.1 ± 0.2	
11a	5'-d(P_yCG C G CG)-3'<br>3'-d(GC G C GC)-5'	B-DNA	53.5 ± 0.3	-72.3 ± 1.5	-198.0 ± 2.5	-10.9 ± 0.1	+0.1
11b	m^5<br>5'-d(P_yCG **C** G CG)-3'<br>3'-d(GC G **C** GC)-5'<br>m^5	Z-DNA	55.6 ± 0.5	-52.8 ± 2.2	-135.4 ± 3.0	-10.8 ± 0.2	
11a*	5'-d(P_yCG C G CG)-3'<br>3'-d(GC G C GC)-5'	B-DNA	51.8 ± 0.3	-69.8 ± 1.5	-190.6 ± 2.5	-10.7 ± 0.1	-0.9
11b*	m^5<br>5'-d(P_yCG **C** G CG)-3'<br>3'-d(GC G **C** GC)-5'<br>m^5	B-DNA	54.2 ± 0.5	-67.1 ± 1.0	-178.9 ± 3.0	-11.6 ± 0.2	

[a]T_m values were determined by UV-based melting analysis at 5 μM total strand concentration in 10 mM sodium phosphate buffer (pH 7.4) containing 150 mM NaCl and 20 mM $MgCl_2$. T_ms reported are the averages of at least six measurements.

[b]Δ*H*° and Δ*S*° values were determined from 1/T_m *versus* ln(strand concentration) plot, assuming a two-state process.

*UV measurements were performed in the absence of $MgCl_2$; under these salt conditions, B-Z structural conversion was not observed for both **7a*** and **11a***.

Table S4. Effects of m^5C modification position on terminal sugar pucker conformation. The $^3J_{H1'–H2'}$ values for the 5'- and 3'-terminal residues of each sequence are indicated in red and green, respectively. For 6-mer CpG duplexes, terminal sugar switch occurs only when the third cytosine residue (C3) is methylated (*i.e.* **1b**), and not when the first (**1c**) or second (**1d**) cytosine residue is methylated. Similarly for 12-mer CpG duplexes, m^5C-induced sugar switch is observed only when methylation is on C3 (**2b**), and not on C1 (**2c**) or C5 (**2d**), suggesting that the structural effect of m^5C is highly-dependent on the position of m^5C methylation. The terminal residues of m^5C-probe **9a** continue to adopt a C3'-*endo* puckering despite a replacement of the RNA backbone of **1a** with 2'-*O*-methyl backbone and the introduction of four relatively bulky 6-phenylpyrrolocytosine (^{P}C) fluorophores. Consistent with our probe design, m^5C methylation of probe **9a** triggered a C3'-*endo* to C2'-*endo* pucker switch in both 5'- and 3'-terminal residues (**9c**).

Entry	Sequences	Conformations	$^3J_{H1'-H2'}$ (Hz)[a] (5'-residue)	Sugar pucker mode	$^3J_{H1'-H2'}$ (Hz)[a] (3'-residue)	Sugar pucker mode
**1a**	5'-r(CG C G CG)-3'<br>3'-r(GC G C GC)-5'	A-RNA	1.1	C3'-*endo*	2.0	C3'-*endo*
**1b**	m^5<br>5'-r(CG C G CG)-3'<br>3'-r(GC G C GC)-5'<br>m^5	A-RNA	**8.2**	**C2'-*endo***	**6.0**	**C2'-*endo***
**1c**	m^5<br>5'-r(CG C G CG)-3'<br>3'-r(GC G C GC)-5'<br>m^5	A-RNA	1.3	C3'-*endo*	1.5	C3'-*endo*
**1d**	m^5<br>5'-r(G C G C G C)-3'<br>3'-r(C G C G C G)-5'<br>m^5	A-RNA	1.7	C3'-*endo*	2.1	C3'-*endo*
**2a**	5'-r(CG C GCGCGC G CG)-3'<br>3'-r(GC G CGCGCG C GC)-5'	A-RNA	1.2	C3'-*endo*	1.1	C3'-*endo*
**2b**	m^5<br>5'-r(CG C GCGCGC G CG)-3'<br>3'-r(GC G CGCGCG C GC)-5'<br>m^5	A-RNA	**8.3**	**C2'-*endo***	**7.9**	**C2'-*endo***
**2c**	m^5<br>5'-r(CG C GCGCGC G CG)-3'<br>3'-r(GC G CGCGCG C GC)-5'<br>m^5	A-RNA	1.2	C3'-*endo*	1.3	C3'-*endo*
**2d**	m^5<br>5'-r(CG C G C GCGCGCG)-3'<br>3'-r(GC G CGCG C GCGC)-5'<br>m^5	A-RNA	1.5	C3'-*endo*	1.9	C3'-*endo*
**9a**	2'-OMe(^{P}C G C G^{P}C G)<br>2'-OMe(G^{P}C G C G^{P}C)	A-RNA	1.3	C3'-*endo*	2.2	C3'-*endo*
**9c**	m^5<br>2'-OMe(^{P}C G C G^{P}C G)<br>2'-OMe(G^{P}C G C G^{P}C)<br>m^5	A-RNA	**8.0**	**C2'-*endo***	**8.1**	**C2'-*endo***

[a]Observed at 25 °C in D_2O.

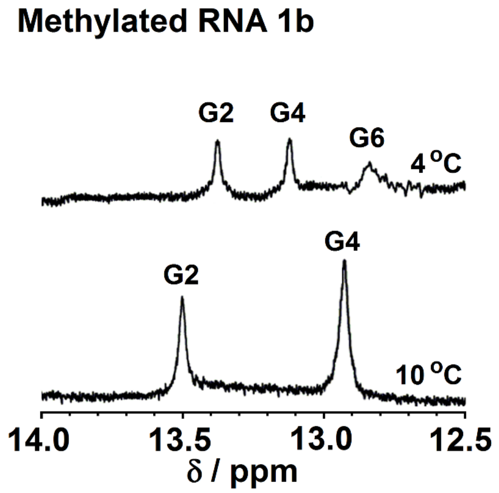

Figure S1. Representative ^{1}H imino NMR spectra of methylated RNA duplex r(CG-**m⁵C**-GCG)$_2$ **1b** in 9:1 buffer/D_2O solvent mix acquired at 4 °C (top) and 10 °C (bottom). The buffer used was 10 mM sodium phosphate buffer (pH 7.4) containing 150 mM NaCl and 20 mM $MgCl_2$. The G6 imino resonance could not be detected at 10 °C, presumably due to ‘fraying’ of the terminal base-pairs.

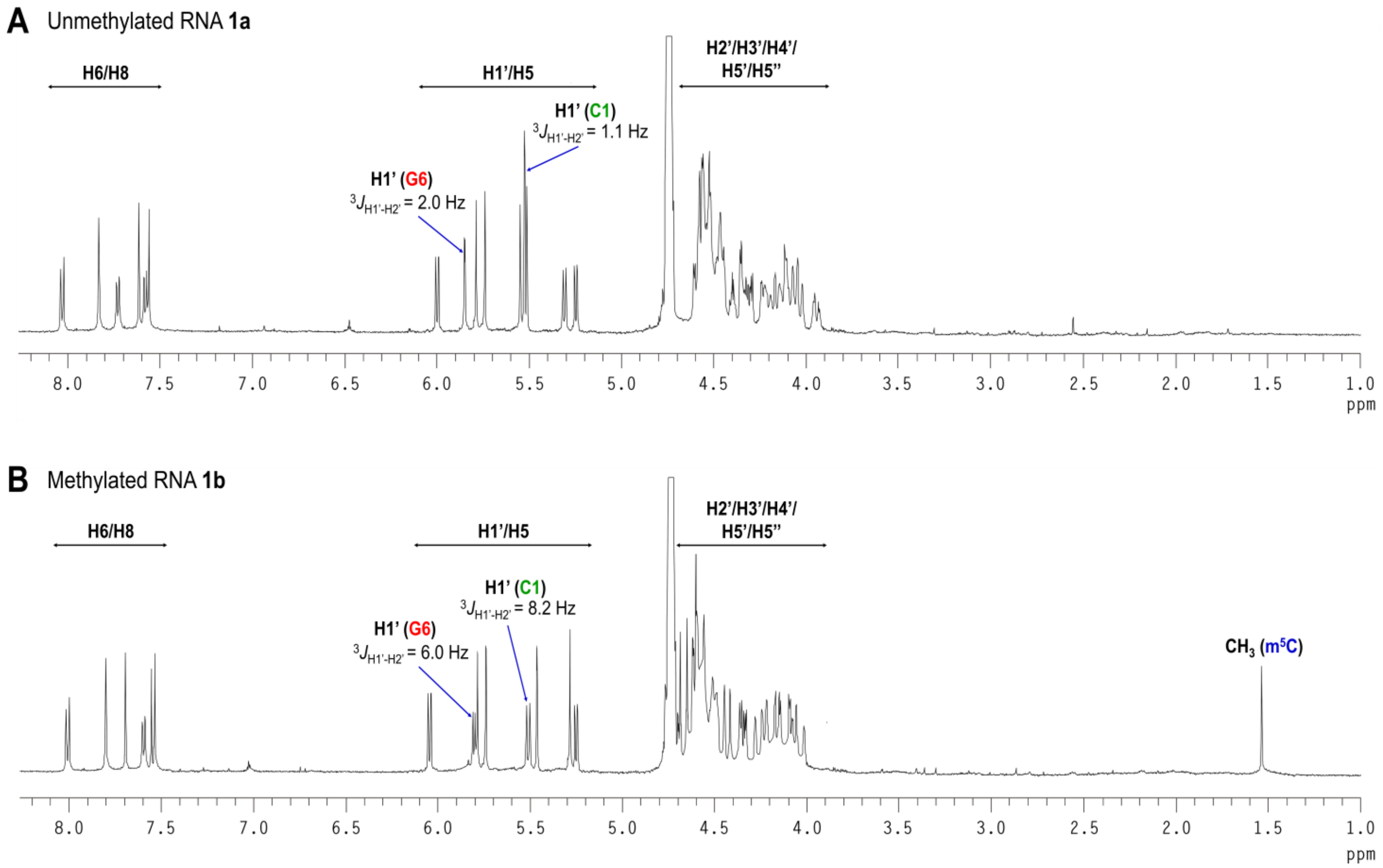

Figure S2. Representative 1D ^{1}H NMR spectra of (A) CpG RNA duplex r(CG-C-GCG)$_2$ **1a** and (B) its methylated counterpart d(CG-**m^{5}C**-GCG)$_2$ **1b**. NMR samples contained 2-3 mM oligos in 300 µL D_2O; spectra were acquired at 25 °C. The coupling constants between sugar protons H1′ and H2′ ($^{3}J_{H1'–H2'}$) for the terminal residues, C1 and G6, are shown. In the absence of m^{5}C methylation, both C1 (δ 5.53) and G6 (δ 5.85) exhibit a C3'-*endo* sugar puckering, with $^{3}J_{H1'–H2'}$ values of 1.1Hz and 2 Hz, respectively. Upon m^{5}C methylation, however, C1 undergoes an unusual sugar pucker switch to C2′-*endo* mode ($^{3}J_{H1'–H2'}$ = 8.2 Hz; δ 5.51), whilst G6 assume an intermediate conformation between C3′-*endo* and C2′-*endo*, with a more C2'-*endo* like characteristics ($^{3}J_{H1'–H2'}$ = 6.0 Hz; δ 5.81). The results demonstrated that m^{5}C methylation can directly affect the terminal sugar pucker conformation of CpG RNA duplexes.

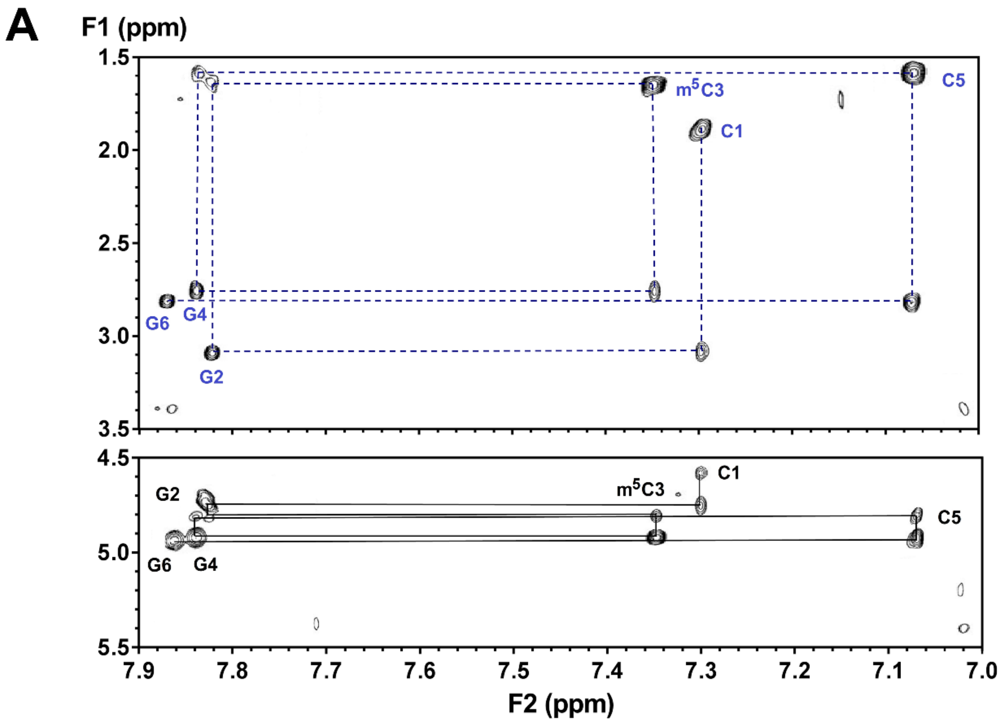

B

Residues	Base-sugar proton distance (Å)[a]	
	H6/H8-H2'	H6/H8-H3'
**C1**	2.2	4.0
**G2**	3.6	2.4
**m^{5}C3**	2.1	4.1
**G4**	3.7	2.5
**C5**	2.1	4.0
**G6**	3.6	2.5

Figure S3. Representative 2D NOESY spectrum of the H2'/H3'-aromatic region of CpG DNA duplex d(CG-**m^{5}C**-GCG)$_2$ **7b** in D_2O at 25 °C (τ_m = 150 ms) showing the sequential assignments of all bases. (A) There are clear NOE cross-peaks between base protons H6/H8 and sugar protons H2' and H3'. The H6/8-H2' NOE connectivity pathways are indicated in blue dashed-line (top), whilst the H6/8-H3' NOE connectivity pathways are shown in black solid line (bottom) (B) The intranucleotide base-sugar distances for **7b** (calculated based on the intensity of NOEs between the base and sugar protons) revealed a C2'-*endo* sugar puckering for all cytosine residues and a C3'-*endo* orientation for all guanosine residues, which is characteristic of Z-DNA structure.

A

Total strand conc/ µM	T_m/ °C	
	7a	7b
30	47.6	64.0
20	46.0	62.3
15	45.7	61.0
10	44.5	59.3
5	43.8	53.4
2	37.1	52.7
1	34.9	49.9

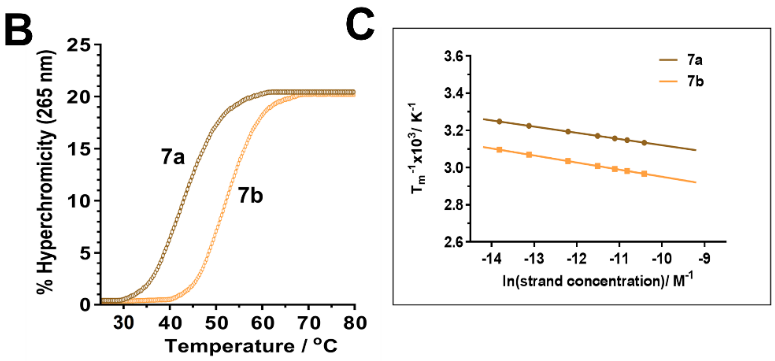

D

Entry	Sequences	Conformations	T_m (°C) (at 5 µM)[a]	$\Delta H°$ (kcal/mol)[b]	$\Delta S°$ (cal/mol/K)[b]	$\Delta G°_{310}$ (kcal/mol)	$\Delta\Delta G°_{310}$ (kcal/mol)[c]
7a	5'-d(CG C G CG)-3'<br>3'-d(GC G C GC)-5'	B-DNA	43.8 ± 0.2	-59.5 ± 2.1	-165.7 ± 2.5	-8.1 ± 0.1	-2.5
7b	5'-d(CG m^5**C** G CG)-3'<br>3'-d(GC G m^5**C** GC)-5'	Z-DNA	53.4 ± 0.2	-52.2 ± 1.7	-134.1 ± 4.6	-10.6 ± 0.2	

Figure S4. UV-based melting analysis of CpG DNA duplex **7a** and its m^5C-methylated counterpart **7b**. (A-B) The UV melting transitions of **7a** (brown line) and **7b** (orange line) were measured at total strand concentrations between 1-30 µM under physiologically-relevant conditions (10 mM sodium phosphate buffer (pH 7.4) containing 150 mM NaCl and 20 mM $MgCl_2$). (C) Van't Hoff analysis of **7a** and **7b** revealed concentration-dependent T_ms, implying that both sequences exist predominantly as bimolecular duplex structures under our experimental conditions. CD analysis revealed a B-DNA and Z-DNA structure for **7a** and **7b**, respectively (see Figure 3). (D) The thermodynamic data were derived from $1/T_m$ *versus* ln(strand concentration) plot, assuming a two-state process. m^5C methylation increases the stability of **7a** by ~2.5 kcal/mol. Moreover, m^5C-induced B-Z helicity change is primarily an entropy-driven process, as judged by the more favourable entropic contributions ($\Delta\Delta S°_{\mathbf{7a}\rightarrow\mathbf{7b}}$ = 31.6 cal/mol/K) which counteracts the enthalpy cost ($\Delta\Delta H°_{\mathbf{7a}\rightarrow\mathbf{7b}}$ = 7.3 kcal/mol).

A

Total strand conc/ µM	T_m/ °C	
	7a*	7b*
30	46.1	52.5
20	45.5	51.0
15	44.9	49.9
10	43.7	48.4
5	42.3	45.7
2	37.1	42.7
1	34.8	40.3

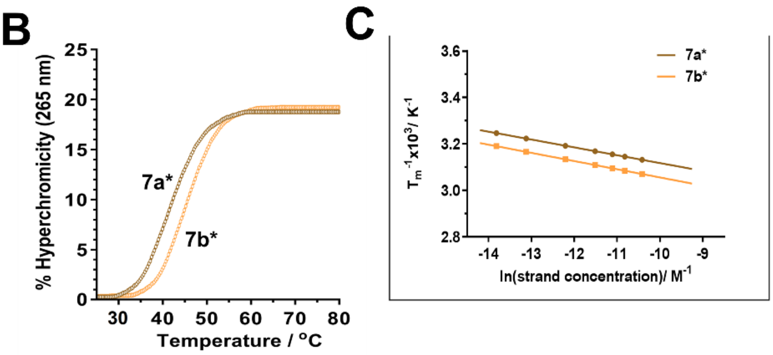

D

Entry	Sequences	Conformations	T_m (°C) (at 5 µM)[a]	$\Delta H°$ (kcal/mol)[b]	$\Delta S°$ (cal/mol/K)[b]	$\Delta G°_{310}$ (kcal/mol)	$\Delta\Delta G°_{310}$ (kcal/mol)[c]
7a*	5'-d(CG C G CG)-3'<br>3'-d(GC G C GC)-5'	B-DNA	42.3 ± 0.3	-58.9 ± 1.3	-163.8 ± 2.5	-8.1 ± 0.2	-1.0
7b*	m5<br>5'-d(CG C G CG)-3'<br>3'-d(GC G C GC)-5'<br>m5	B-DNA	45.7 ± 0.3	-56.4 ± 1.5	-152.5 ± 4.6	-9.1 ± 0.2	

Figure S5. UV-based melting analysis of CpG DNA duplex **7a*** and its m^{5}C-methylated counterpart **7b*** *in the absence of Z-conformation inducer MgCl$_2$*. (A-B) The UV melting transitions of **7a*** (brown line) and **7b*** (orange line) were measured at total strand concentrations between 1-30 µM under physiologically-relevant conditions (10 mM sodium phosphate buffer (pH 7.4) containing 150 mM NaCl and 20 mM MgCl$_2$). (C) Van't Hoff analysis of **7a*** and **7b*** revealed concentration-dependent T_ms, implying that both sequences exist predominantly as bimolecular duplex structures under our experimental conditions. CD analysis revealed a B-DNA structure for both **7a*** and **7b*** (see Figure S6). (D) The thermodynamic data were derived from 1/T_m *versus* ln(strand concentration) plot, assuming a two-state process. m^{5}C methylation did not induce B-Z conformational change in **7a*** in the absence of MgCl$_2$, hence any free energy differences observed between **7a*** and **7b*** is largely attributed to duplex stabilisation by m^{5}C ($\Delta\Delta G°_{310}$ ~0.5 kcal/mol per m^{5}C); m^{5}C methylation also increases the melting temperatures (T_m) of **7a*** by ~1.7 °C per m^{5}C.

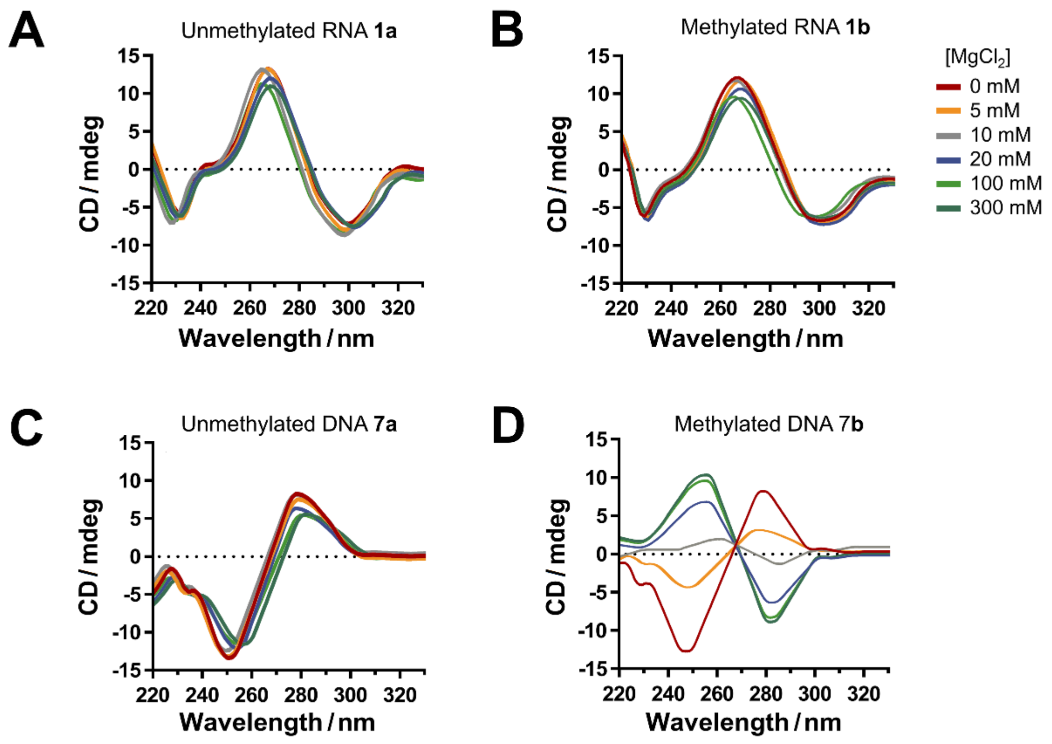

Figure S6. CD analyses of (A) CpG RNA duplex **1a** and (B) its m^{5}C-methylated counterpart **1b**, as well as (C) CpG DNA duplex **7a** and (D) its m^{5}C-methylated counterpart **7b** at a range of $MgCl_2$ concentrations (0-300 mM). The RNA duplexes **1a** and **1b** maintained an A-type double helical structure at all $MgCl_2$ concentrations tested. Intriguingly, the methylated RNA **1b** could not be induced to form Z-RNA even at $MgCl_2$ concentration as high as 300 mM. On the contrary, methylated DNA duplex **7b** readily undergoes B-Z structural transformation, as evidenced by an inversion of the CD spectrum; the midpoint $MgCl_2$ concentration for B-Z transition is ~10 mM.

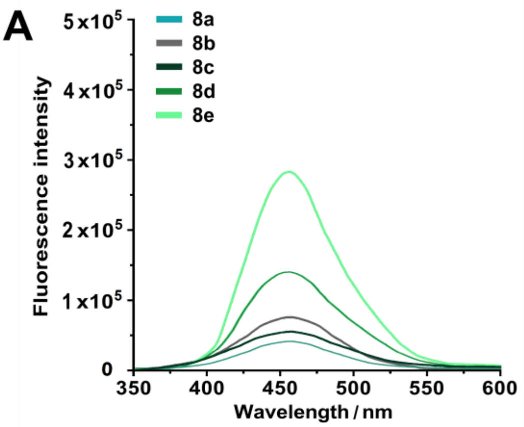

B

Entry	Sequences	Quantum yield (Φ_F)	Fold change
8a	2'-OMe(^{P}CG C G C G)<br>2'-OMe(GC G C G C)	0.03 ± 0.01	--
8b	2'-OMe(^{P}CG $\overset{m^5}{C}$ G C G)<br>2'-OMe(GC G C G C)	0.06 ± 0.01	2.0
8c	2'-OMe(^{P}CG C G C G)<br>2'-OMe(GC G C G^{P}C)	0.04 ± 0.02	--
8d	2'-OMe(^{P}CG $\overset{m^5}{C}$ G C G)<br>2'-OMe(GC G C G^{P}C)	0.11 ± 0.02	2.8
8e	2'-OMe(^{P}CG $\overset{m^5}{C}$ G C G)<br>2'-OMe(GC G $\underset{m^5}{C}$ G^{P}C)	0.21 ± 0.03	5.3

Figure S7. Effects of m^5C methylation levels on the (A) fluorescence light-up response and (B) fluorescence quantum yield of RNA probes **8a** and **8c**. The fluorescence emission spectra (λ_{ex} 360 nm; λ_{em} 465 nm) of the probes were recorded at 5 µM strand concentration under physiologically-relevant conditions (10 mM sodium phosphate buffer (pH 7.4) containing 150 mM NaCl and 20 mM $MgCl_2$, 37 °C). Probes **8a** displays weak starting fluorescence (Φ_F 0.03), but also give unremarkable fluorescence increase upon methylation (**8b**; Φ_F 0.06). The incorporation of an additional 5'-terminal ^{P}C in the opposite strand to produce probe **8c** (Φ_F 0.04) led to a significant improvement in fluorescence response. In particular, the singly-methylated probe **8d** now yields a 2.8-fold increase in fluorescence intensity (Φ_F 0.11), whilst the doubly-methylated probe **8e** gives a 5.3-fold increase in signal (Φ_F 0.21). This represents approximately a doubling of fluorescence emission, thus the probe fluorescence increases proportionately with m^5C methylation levels.

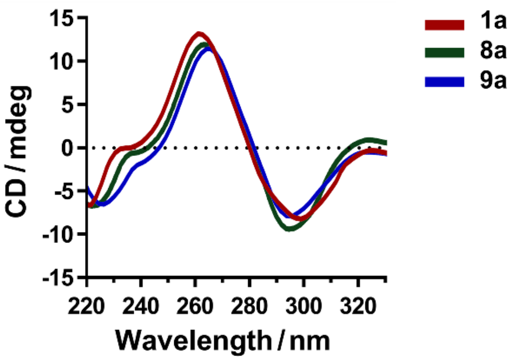

Figure S8. Superimposition of the CD spectrum of m^{5}C-probe **9a** with that of its parent probes **8a** and **1a** revealed no significant change in their secondary structures. All duplexes adopt an A-form RNA structure. Thus the presence of four relatively bulky ^{p}C residues has negligible effects on the overall secondary structure of **9a**. This result is consistent with the ability of ^{p}C to base-pair with guanine in a manner analogous to cytosine.

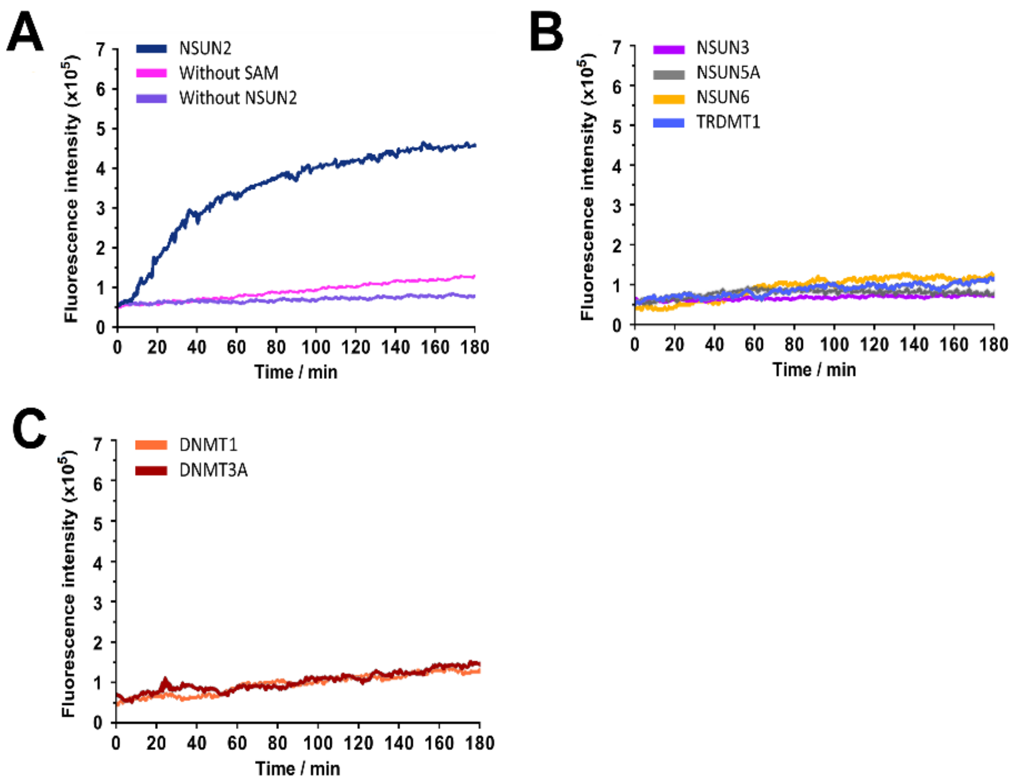

Figure S9. Fluorescence activation of m^{5}C-probe **9a** was specifically triggered by NSUN2. (A) Time-course fluorescence analyses (λ_{ex} 360 nm; λ_{em} 465 nm) of probe **9a** (5 µM) in the presence of NUSN2 (0.5 µM) gave a bright blue fluorescence after a short 30-minute incubation; maximum intensity was reached after 3 h, giving ~8 fold increase in fluorescence signal. No fluorescence light-up response was detected in the absence of NSUN2 or methyl donor SAM. (B) There was no appreciable change in the fluorescence intensity (λ_{em} 465 nm) when the probe was exposed to other human RNA:m^{5}C MTases, including TRDMT1 (tRNA C_{38}) and three structurally-related homologues NSUN3 (mt tRNA C_{34}), NSUN5A (28S rRNA C_{3761}), and NSUN6 (tRNA C_{72}). Their reported physiological substrates were highlighted in blue. (C) There was also no significant fluorescence response with human DNA:m^{5}C MTases, namely DNMT1 (a maintenance MTase) and DNMT3A (a *de novo* MTase). Hence probe **9a** is highly-selective for NSUN2 over other RNA/DNA:m^{5}C MTases.

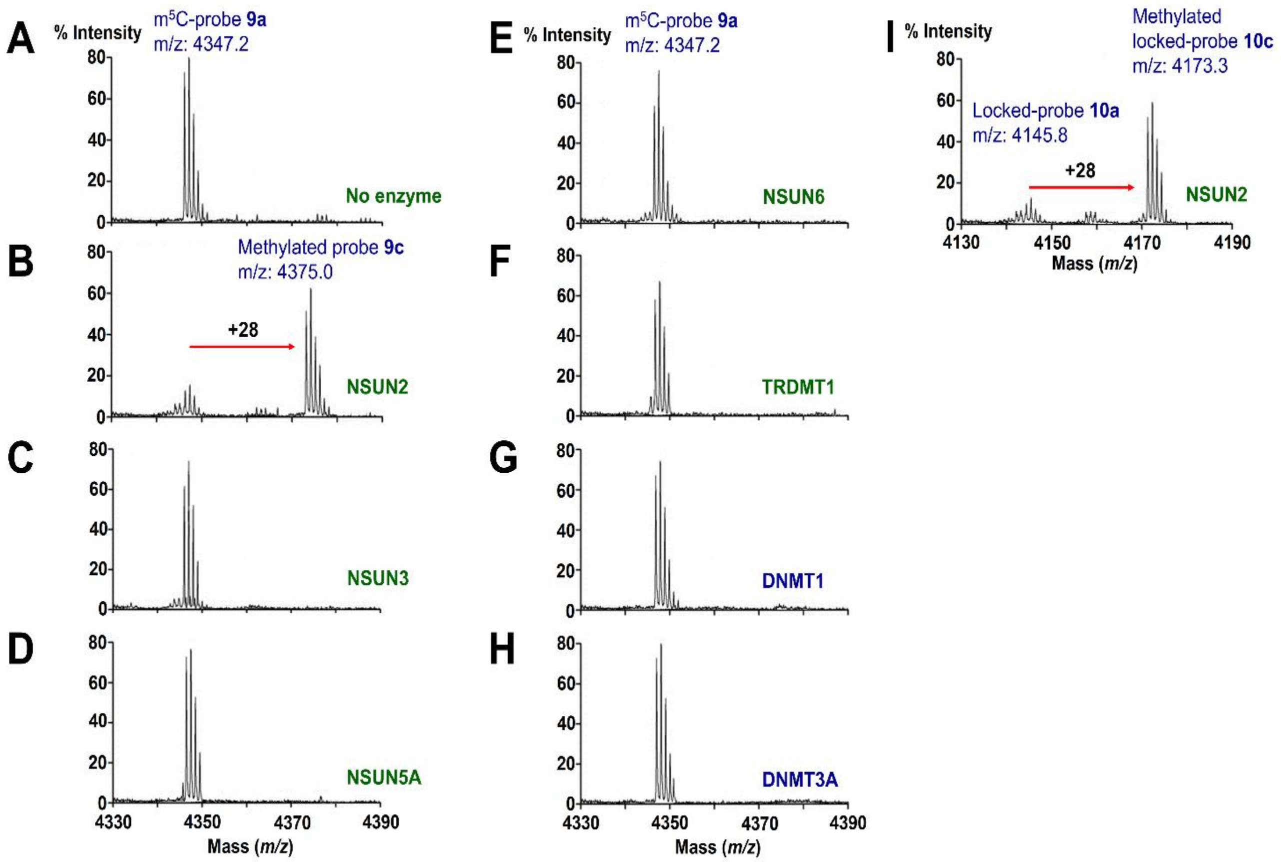

Figure S10. MALDI-TOF MS assays of the m^{5}C-probe **9a** against a panel of human RNA/DNA:m^{5}C MTases. (A-B) Incubation of probe **9a** (5 µM; m/z 4347.2) with NSUN2 (1 µM) and methyl donor SAM (200 µM) for 3 h led to significant formation of methylated product (m/z 4375.0). Under similar assay conditions, there was negligible methylated product formation with (C) NSUN3, (D) NSUN5A, (E) NSUN6, (F) TRDMT1, (G) DNMT1, and (H) DNMT3A, even after a prolonged 8 h-incubation. (I) As with probe **9a**, the locked-probe **10a** (m/z 4145.8) can also be readily methylated by NSUN2 to give the corresponding methylated product (m/z 4173.3), however this was not accompanied with fluorescence light-up response, suggesting that fluorescence activation is likely dependent on a change in sugar puckering of the ^{P}C residues.

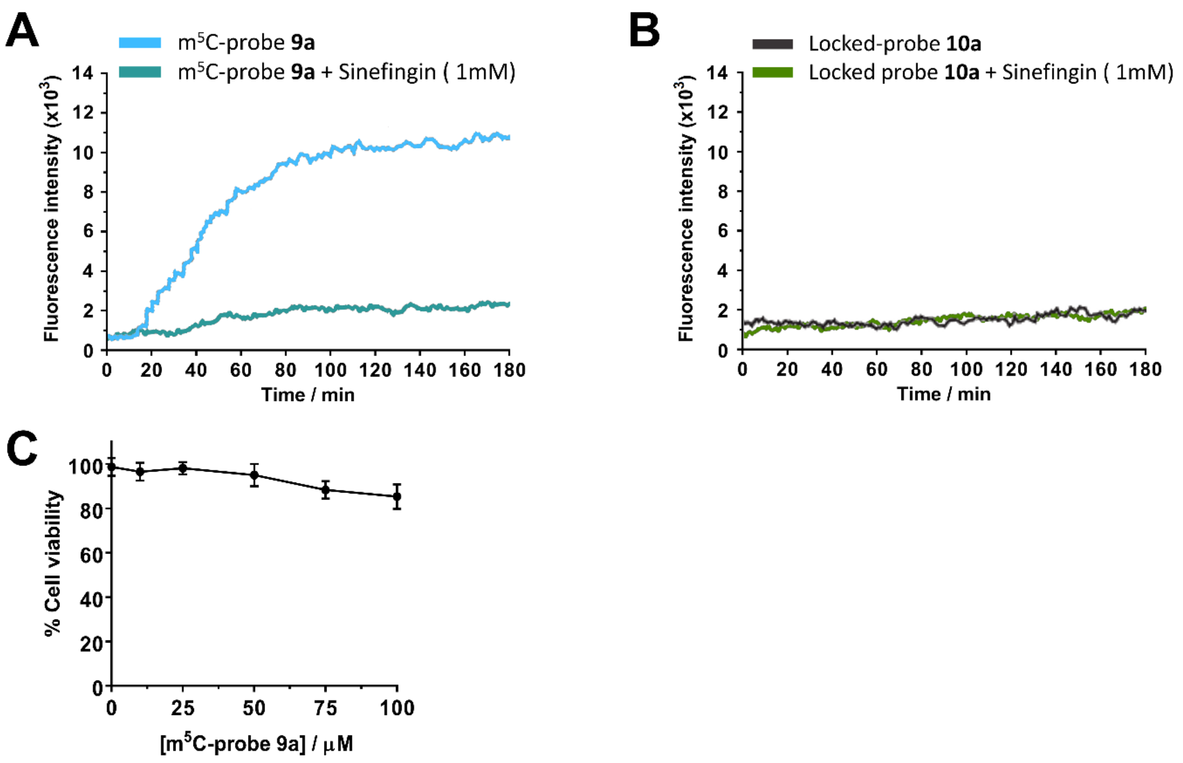

Figure S11. The m^{5}C-probe **9a** demonstrates good cellular stability and low cytotoxicity. (A) HeLa cell lysate experiments to investigate fluorescence due to non-specific degradation of m^{5}C-probe **9a**. Probe **9a** (10 µM) was incubated with HeLa cell lysate which had been spiked with NSUN2 inhibitor sinefungin (1 mM; green line) at 37 °C. Under such high concentration of sinefungin, most, if not all, NSUN2 activity would have been inhibited, thus any increase in fluorescence signal could be attributed to probe degradation or other NSUN2-*independent* mechanisms. We observed no obvious increase in fluorescence signal at 465 nm (λ_{ex} 360 nm) over a time-course of 3 h, suggesting no significant probe degradation and/or activation by other non-specific mechanisms. In cell lysate that had not been spiked with sinefungin (blue line), however, we observed a significant increase in fluorescence within 30 min of incubation. The methylated probe that was generated remained strongly emissive for at least 3 h, with no perceptible decline in intensity, suggesting that the probe has good photostability. (B) A repeat of the cell lysate experiment using locked-probe **10a** (which is not activated by NSUN2) gave very little or no increase in fluorescence in HeLa cell lysate, both in the absence and presence of 1 mM sinefungin. These results suggest that probe degradation do not interfere with the performance of the m^{5}C-probe **9a** significantly, at least not within the time frame of our cell-based assay, which is typically 1 h. (C) MTT cytotoxicity assay showed that the probe is well tolerated by HeLa cells, with > 80% of the cells remaining viable after treatment with 40 µM probe for 24 h. Data are expressed as mean ± SD of three biological replicates.

A

Total strand conc/ μM	T_m/ °C	
	11a	11b
30	57.5	65.1
20	56.3	63.3
15	55.4	62.1
10	54.2	60.4
5	53.5	55.6
2	49.6	53.8
1	47.6	51.0

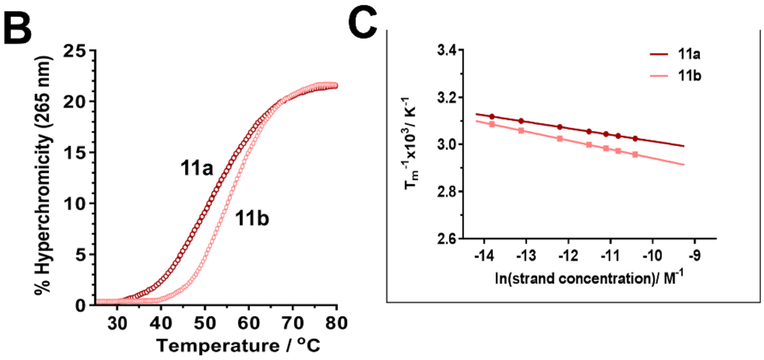

D

Entry	Sequences	Conformations	T_m (°C) (at 5 μM)[a]	$\Delta H°$ (kcal/mol)[b]	$\Delta S°$ (cal/mol/K)[b]	$\Delta G°_{310}$ (kcal/mol)	$\Delta\Delta G°_{310}$ (kcal/mol)[c]
11a	5'-d(PyCG C G CG)-3'<br>3'-d(GC G C GC)-5'	B-DNA	53.5 ± 0.3	-72.3 ± 1.5	-198.0 ± 2.5	-10.9 ± 0.1	+0.1
11b	5'-d(PyCG $\overset{m^5}{C}$ G CG)-3'<br>3'-d(GC G $\underset{m^5}{C}$ GC)-5'	Z-DNA	55.6 ± 0.5	-52.8 ± 2.2	-135.4 ± 3.0	-10.8 ± 0.2	

Figure S12. UV-based melting analysis of DNA m^{5}C-probe **11a** and its m^{5}C-methylated counterpart **11b**. (A-B) The UV melting transitions of **11a** (red line) and **11b** (pink line) were measured at total strand concentrations between 1-30 μM under physiologically-relevant conditions (10 mM sodium phosphate buffer (pH 7.4) containing 150 mM NaCl and 20 mM $MgCl_2$). (C) Van't Hoff analysis of **11a** and **11b** revealed concentration-dependent T_ms, implying that both probes exist predominantly as bimolecular duplex structures under our experimental conditions. CD analysis revealed a B-DNA and Z-DNA structure for **11a** and **11b**, respectively (see Figure 8). (D) The thermodynamic data were derived from $1/T_m$ *versus* ln(strand concentration) plot, assuming a two-state process. P_y is able to stack readily at the 5'-end of probe **11a**, giving an end-stacking stabilisation of ~2.8 kcal/mol (compare the difference in duplex stability between **11a** and **7a**). Upon m^{5}C methylation, however, end-stacking interaction is completely abolished (negligible difference in stability between **11b** and **7b**; $\Delta\Delta G°_{310}$ ~0.2 kcal/mol).

A

Total strand conc/ µM	T_m/°C	
	11a*	11b*
30	57.3	63.0
20	56.0	61.6
15	55.1	60.7
10	53.9	59.3
5	51.8	54.2
2	49.1	53.1
1	47.0	51.9

B

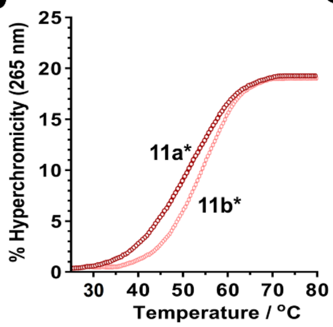

C

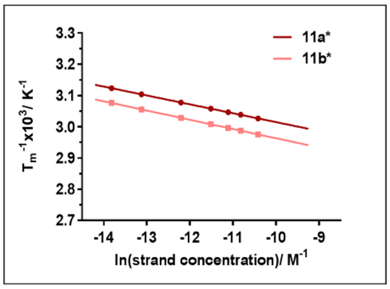

D

Entry	Sequences	Conformations	T_m (°C) (at 5 µM)[a]	$\Delta H°$ (kcal/mol)[b]	$\Delta S°$ (cal/mol/K)[b]	$\Delta G°_{310}$ (kcal/mol)	$\Delta\Delta G°_{310}$ (kcal/mol)[c]
11a*	5'-d(PyCG C G CG)-3'<br>3'-d(GC G C GC)-5'	B-DNA	51.8 ± 0.3	-69.8 ± 1.5	-190.6 ± 2.5	-10.7 ± 0.1	-0.9
11b*	m5<br>5'-d(PyCG C G CG)-3'<br>3'-d(GC G C GC)-5'<br>m5	B-DNA	54.2 ± 0.5	-67.1 ± 1.0	-178.9 ± 3.0	-11.6 ± 0.2	

Figure S13. UV-based melting analysis of DNA m^{5}C-probe **11a*** and its m^{5}C-methylated counterpart **11b*** *in the absence of Z-conformation inducer* $MgCl_2$. (A-B) The UV melting transitions of **11a*** (red line) and **11b*** (pink line) were measured at total strand concentrations between 1-30 µM under physiologically-relevant conditions (10 mM sodium phosphate buffer (pH 7.4) containing 150 mM NaCl and 20 mM $MgCl_2$). (C) Van't Hoff analysis of **11a*** and **11b*** revealed concentration-dependent T_ms, implying that both probes exist predominantly as bimolecular duplex structures under our experimental conditions. CD analysis revealed a B-DNA structure for **11b*** (see Figure 8). (D) The thermodynamic data were derived from $1/T_m$ *versus* ln(strand concentration) plot, assuming a two-state process. m^{5}C methylation did not induce B-Z conformational change in **11a*** in the absence of $MgCl_2$, hence any free energy differences observed between **11a*** and **11b*** is largely attributed to duplex stabilisation by m^{5}C ($\Delta\Delta G°_{310}$ ~0.45 kcal/mol per m^{5}C); m^{5}C methylation also increases the melting temperatures (T_m) of **11a*** by ~1.2 °C per m^{5}C.

Materials and Methods

Preparation of 2'-*O*-methyl RNA and DNA m^{5}C-probes

All oligonucleotide probes used in this study were synthesised on an Applied Biosystems 394 DNA/RNA synthesiser using standard β-cyanoethyl phosphoramidite chemistry. All synthesiser reagents, 2'-*O*-methyl phosphoramidites, and 2'-deoxy phosphoramidites were purchased from Glen Research. 2'-*O*-methyl 6-phenylpyrrolocytidine phosphoramidite[1,2] and pyrene deoxynucleoside phosphoramidite[3] were synthesised according to literature procedure. The oligonucleotides were synthesised on a CPG solid support using a standard 1 μmole phosphoramidite cycle of acid-catalysed detritylation, coupling, capping, and iodine oxidation. Coupling of 2'-*O*-methyl 6-phenylpyrrolocytidine and other modified monomers e.g. 5-methylcytidine (m^{5}C) and pyrene deoxynucleoside (P_y) was achieved *via* coupling reagent 5-(ethylthio)-1*H*-tetrazole using an extended coupling time of 10 min. Cleavage of the oligonucleotides from the solid support was performed by treatment with an anhydrous solution of 2 M ammonia in MeOH for 60 h at room temperature. The crude product was then lyophilised and purified by reverse-phase HPLC using the Waters XBridge OST C18 column (2.5 micron, 10 mm × 50 mm). HPLC solvents used were: solvent A (100 mM triethylammonium acetate buffer, pH 6.5 with 5% acetonitrile) and solvent B (100 mM triethylammonium acetate buffer, pH 6.5 with 15% acetonitrile) with a flow rate of 5 mL/ min. All purified oligonucleotides were characterised by MALDI-TOF MS and capillary gel electrophoresis, and were found to be at least 95% pure. The MALDI-TOF MS data for all probes investigated in this study are summarised in Tables S1 and S2.

DNA/RNA methyltransferase inhibitors

All DNA/RNA MTase inhibitors (sinefungin, *S*-adenosyl-L-homocysteine (SAH), adenosine and homocysteine) investigated in this study were purchased from Sigma-Aldrich and used without further purification.

Human m^{5}C:RNA MTases (NSUN2, NSUN3, NSUN5A and NSUN6 proteins)

Full-length human NSUN2 (containing *C*-terminal MYC/DDK tag; TP314459) and full-length human NSUN6 (containing *C*-terminal MYC/DDK tag; TP307817) proteins were purchased from Origene Technologies Inc., USA.

Full-length human NSUN3 (containing *N*-terminal GST tag; ab163752), full-length human NSUN5A (containing *N*-terminal GST tag; ab163181), and full-length human TRDMT1 (formerly DNMT2; containing *N*-terminal GST tag; ab124566) proteins were purchased from abcam plc.,UK.

Human m^5C:DNA MTases (DNMT1 and DNMT3A proteins)

Human DNMT1 protein encompassing residues 501-1632 containing *N*-terminal His-DDDDK tag was purchased from abcam plc. UK (ab198109).

Full-length human DNMT3A protein containing *N*-terminal His-DDDDK tag was purchased from abcam plc. UK (ab170408).

Circular dichroism (CD) spectroscopy

The CD spectra for the duplexes were obtained using a JASCO J-810 spectropolarimeter, as previously described.[4] Measurements were carried out at 25 °C with 5 μM probes in a 10 mM sodium phosphate buffer (pH 7.4) containing 150 mM NaCl, and varying $MgCl_2$ concentrations (0, 5, 10, 20, 100, and 300 mM). The concentrations were determined by UV absorbance at 260 nm (A_{260}) using a NanoDrop ND-1000 UV-Visible Spectrophotometer. The oligo solutions were first heated to 90 °C for 5 min, and re-annealed by slow cooling to 4 °C at a rate of 1 °C/min. CD spectra were then recorded in quartz cuvettes (path length 1 mm, 400 μL) from 220 nm to 330 nm at 25 °C using a 10 nm/min scan speed, a spectral band width of 1 nm and a time constant of 4 s. All the spectra were subtracted with the buffer blank and smoothed using the Savitsky-Golay algorithm (polynomial order 10).

UV-based thermal denaturation experiments

The melting of each duplex (**1a, 1b, 7a, 7b, 11a** and **11b**) was performed on a Cary 300 UV-Visible Spectrophotometer (Varian) at a total strand concentration of 5 μM (unless otherwise stated) in 10 mM sodium phosphate buffer (pH 7.4), 150 mM NaCl and 20 mM $MgCl_2$. Absorbance versus temperature profiles were recorded at 260 nm. The samples were first denatured by heating to 95 °C at 10 °C/min, followed by slow cooling to 25 °C at 0.4 °C/min to ensure a complete annealing of the strands. The melting transitions were then monitored by heating to 95 °C at 0.4 °C/min. To increase the accuracy of measurements, the sixth position was used to record the temperature data points by placing a temperature probe directly in the cuvette. Each oligo was measured at seven different concentrations (1, 2, 5, 10, 15, 20 and 30 μM), and a total of six independent melting transitions were measured for each concentration. The T_m values were determined by the Varian Cary Software using the baseline (alpha) method, which is defined as the point when the mole fraction of duplex was equal to 0.5. The melting transitions were generally found to be reproducible for all oligos studied.

Thermodynamic parameters derived from UV-melting experiments

The thermodynamic data for methylated and unmethylated (**1a, 1b, 7a, 7b, 11a** and **11b**) were analysed as previously described, with modifications.[5,6] The melting transitions for duplex structures were assumed to proceed in a two-state manner, and to obey the van't Hoff's equation below.

$$\frac{1}{T_{\mathrm{m}}} = \frac{R}{\Delta_b H^{\circ}} \ln c_T + \frac{\Delta_b S^{\circ}}{\Delta_b H^{\circ}}$$

A plot of $1/T_{\mathrm{m}}$ versus ln(total strand concentration) gives a straight line, where the slope is $\mathrm{R}/\Delta H°$ and the y-intercept is $\Delta S°/\Delta H°$. Data were fitted using linear least-squares minimisation using GraphPad Prism. The free Gibbs energy ($\Delta G°$) were calculated at 37 °C (310.15 K) using the following equation. The results are summarised in Supplementary Table S3.

$$\Delta_b G^{\circ} = \Delta_b H^{\circ} - T\Delta_b S^{\circ}$$

Determination of fluorescence quantum yields of m^{5}C-probes

The fluorescence quantum yields (Φ_F) of 6-phenylpyrrolocytosine (^{P}C)-labelled 2'-*O*-methyl RNA probes **8a-e**, and **9a-c** were determined using previously described methods, with modifications.[7-10] UV absorption of the probes at λ_{ex} 370 nm was measured on a Cary-300 UV-Visible spectrophotometer (Varian Inc.) from 0.1 to 0.03 absorbance units in 0.01 increments. The fluorescence emission spectra were immediately measured afterwards in a Cary Eclipse fluorescent spectrophotometer (Varian Inc.) with an excitation wavelength of 370 nm. 9,10-diphenylanthracene (DPA) in ethanol was used as a reference standard, and the quantum yield was calculated using the equation below. The reported quantum yields are an average of three measurements within ±10%.

$$\Phi_F(\text{probe}) = \Phi_F\,(\text{DPA}) \times \frac{\text{A(probe)}}{\text{A}_{370}\text{(probe)}} \times \frac{1}{\alpha(\text{DPA})} \times \frac{\eta(\text{H}_2\text{O})^2}{\eta(\text{EtOH})^2}$$

Where:

Φ_F(DPA) is the cross-calibrated value for the fluorescence quantum emission yield of 9,10-diphenylanthracene (DPA) in ethanol. Under our experimental conditions, the fluorescence quantum yield of DPA was determined to be 0.93, which is highly consistent with the reported value of 0.95.[7-10]

A(probe) is the area of the fluorescence emission spectra of the sample from 350 to 500 nm

A_{370}(probe) is the absorbance of the sample at λ_{ex} 370 nm

α(DPA) is the slope of the fluorescence emission versus A_{370}(probe) calibration curve for DPA

η(H_2O) and η(EtOH) are the refractive indexes of water (1.333) and ethanol (1.361), respectively.

The fluorescence quantum yields (Φ_F) of pyrene (P_y)-labelled DNA probes **11a-c** were determined as previously described.[7,11] UV absorption of the probes λ_{ex} 340 nm was measured on a Cary-300 UV-Visible spectrophotometer (Varian Inc.) from 0.1 to 0.03 absorbance units in 0.01 increments. The fluorescence emission spectra were immediately measured afterwards in a Cary Eclipse (Varian Inc.) fluorescent spectrophotometer with an excitation wavelength of 340 nm. Pyrenebutanoic acid (PBA) in methanol was used as a reference, and was calculated using the equation below. The reported quantum yields are an average of three measurements within ±10%.

$$\Phi_F(\text{probe}) = \Phi_F\,(\text{PBA}) \times \frac{A(\text{probe})}{A_{340}(\text{probe})} \times \frac{1}{\alpha(\text{PBA})} \times \frac{\eta(H_2O)^2}{\eta(\text{MeOH})^2}$$

Where:

Φ_F(PBA) is the cross-calibrated value for the fluorescence quantum emission yield of pyrenebutanoic acid (PBA) in MeOH. Under our experimental conditions, the fluorescence quantum yield of PBA was determined to be 0.061, which is highly consistent with the reported value of 0.065.[7,8,11]

A(probe) is the area of the fluorescence emission spectra of the sample from 350 to 500 nm

A_{340}(probe) is the absorbance of the sample at λ_{ex} 340 nm

α(PBA) is the slope of the fluorescence emission versus A_{340}(probe) calibration curve for PBA

η(H_2O) and η(MeOH) are the refractive indexes of water (1.333) and methanol (1.328), respectively.

MALDI-TOF MS-based methyltransferase assay

The assay was modified from previously reported method.[12] The assay was performed in triplicate in a final reaction volume of 25 µL. Reaction consisted of substrate (m^5C-probe **9a** or locked probe **10a**; 5 µM), enzyme (1 µM), methyl donor *S*-adenosyl-L-methionine (SAM; 200 µM), in 50 mM HEPES buffer (pH 7.4) containing 150 mM NaCl and 20 mM $MgCl_2$. The reaction was incubated at 37 ºC for 1 h, before 1:1 quenching with 20% v/v formic acid. 1 uL of the diluted assay mixture was then mixed with 1 uL of 3-hydroxpicolinic acid (3-HPA, the MALDI-TOF-MS matrix, Sigma-Aldrich) and spotted onto the MALDI-TOF-MS plate before analysis. The 3-HPA matrix was prepared by mixing 9 parts of 50 mg/mL 3-hydroxpicolinic acid in 50% MeCN/Milli-Q H_2O with 1 part of 50 mg/mL ammonium citrate in Milli-Q H_2O. The percentage methylation was estimated based on the relative intensities of the substrate and the methylated product observed in the mass spectra.

HeLa cell culture

HeLa cells (ATCC) were cultured in Dulbecco's modified Eagle's medium (DMEM; Life Technologies) supplemented with 10% fetal bovine serum (FBS), 100 units/mL penicillin, and 100 μg/mL streptomycin at 37 °C in a humidified incubator containing 5% CO_2 environment.

Small interfering RNA (siRNA) gene silencing

To silence the expression of specific gene in Hela cells transiently, 0.5 x 10^5 cells were plated in each well of a 24-well plate one day prior to transfection with siRNA. The next day, the cells were transfected with either siNSUN2, siNSUN6, siTRDMT1, siDNMT3A or siControl using Lipofectamine RNAiMAX (Invitrogen) following the manufacturer's protocols. The sequences for siRNAs and siControls used are provided below. Unless otherwise indicated, cells were analysed 24 h after transfection, and silencing of gene was monitored by Western blot using the respective antibodies.

siNSUN2: 5'-AGAUGUUAAGAUACUGUUGACCC-3'
siControl: 5'-UUGUUCGAACGUGUCACGUTT-3'
anti-NSUN2 antibodies: purchased from Abcam; ab107262

siNSUN6-1: 5'-UUAGUAACUGAAUCCGUGGUG-3'
siNSUN6-2: 5'-UUCUGGGUCCAAUAACAGGAA-3'
siControl: 5'-UCGUAAGUAAGCGCAACCC-3'
anti-NSUN6 antibodies: purchased from Santa Cruz Biotechnology; sc-393446
siTRDMT1 (formerly siDNMT2): purchased from Santa Cruz Biotechnology, sc-35205
siControl: fluorescein conjugate-A, purchased from Santa Cruz Biotechnology, sc-36869.
anti-TRDMT1 antibodies: purchased from Santa Cruz Biotechnology; sc-365001.

siDNMT3A-1: 5′-UGAGAAAGAGGACAUCUUAtt-3′,
siDNMT3A-2:5′-GGGACAUCUCGCGAUUUCUCGtt-3′
siControl: 5′-AGAUAACAUAGACUGUGAGtt-3′,
anti-DNMT3A antibodies: purchased from Santa Cruz Biotechnology; sc-365769

Gene overexpression

NSUN2. The construction of pcDNA3-Flag vector expressing NSUN2 was previously described.[13] In brief, the full length *NSUN2* gene was amplified by PCR using human HeLa cells cDNA (primers 5'-CCGAATTCAATGGGGCGGCGGTCGCGGGGT-3') and subcloned into the EcoRI-BamHI sites

of mammalian pcDNA3-Flag vector (Invitrogen) to obtain pcDNA3-Flag-NSUN2. The plasmids were then transfected into HeLa cells using the calcium chloride transfection method.

NSUN6. The construction of pcDNA3-Flag vector expressing NSUN6 was previously described.[14] In brief, pcDNA3.1-Flag was constructed by cloning *N*-terminal 3xFlag tag into pcDNA3.1 (Invitrogen) plasmid at Hind III and Kpn I sites. The cDNA encoding *NSUN6* was PCR-amplified by the Phusion DNA polymerase and subcloned into the KpnI and XhoI sites of pcDNA3.1-Flag with CloneExpress II One step Cloning Kit (Vazyme), generating plasmid pcDNA3-Flag-NSUN6. The plasmids were then transfected into HeLa cells using the calcium chloride transfection method. The primer sequences used for vector construction are:
Forward: 5'-gacgatgacgataagggtaccATGTCTATTTTCCCTAAGATATCTTTGAGA-3'
Reverse: 5'-aacgggccctctagactcgagTCATGTGCTTTTGCATTTTACAAA-3'

TRDMT1. The construction of pcDNA3-Flag vector expressing TRDMT1 was previously described.[15] In brief, the full length *TRDMT1* gene was amplified by PCR using human HeLa cells cDNA (Clontech) and subcloned into the EcoRI-BamHI sites of mammalian pcDNA3-Flag vector (Invitrogen) to generate pcDNA3-Flag-TRDMT1. The plasmids were then transfected into HeLa cells using the calcium chloride transfection method. The primer sequences used for vector construction are:
Forward: 5'-GAAGAATTCATGGAGCCCCTGCGG-3'
Reverse: 5'-GGAGGATCCATGGAGCCCCTGCGG-3'

DNMT3A. The construction of pcDNA3-Flag vector expressing DNMT3A was previously described.[16] In brief, the full length *DNMT3A* gene was amplified by PCR using human HeLa cells cDNA (Clontech) and subcloned into the EcoRI-BamHI sites of mammalian pcDNA3-Flag vector (Invitrogen) to generate pcDNA3-Flag-DNMT3A. The plasmids were then transfected into HeLa cells using the calcium chloride transfection method. The primer sequences used for vector construction are:
Forward: 5'-CTCTCGCCTCCAAAGACCACGAT-3'
Reverse: 5'-AACTTTGTGTCGCTACCTCAG-3'

Western blot analysis

Cells were first lysed using RIPA buffer (25 mM Tris-HCl (pH 7.6), 150 mM NaCl, 0.1% SDS, 1% sodium deoxycholate, 1% NP-40 and 1x protease inhibitors (Roche), followed by immunoblotting using standard protocol. In brief, equal quantities of proteins were loaded and separated by sodium dodecyl sulfate-polyacrylamide gel electrophoresis (SDS-PAGE) and transferred onto nitrocellulose

filter membranes (Whatman). After incubation with respective antibodies, the blots were incubated with either horseradish peroxidase (HRP)-conjugated anti-rabbit IgG antibody (Bio-Rad) or HRP-conjugated anti-mouse IgG antibody (Bio-Rad) for 2 h at room temperature. Enhanced chemiluminescence substrates (Luminata Crescendo, EMD Millipore) were then applied, and the signals exposed to autoradiography film. The immunoblots were then quantified by densitometric analyses using ImageJ software. All antibodies used were purchased from commercial sources: anti-NSUN2 (Abcam; ab107262), anti-NSUN6 (Santa Cruz Biotechnology; sc-393446), anti-TRDMT1 (Santa Cruz Biotechnology; sc-365001), anti-DNMT3A (Santa Cruz Biotechnology; sc-365769), anti-Flag (Sigma-Aldrich; F1804), and anti-GAPDH antibody (Abcam; ab9483; loading control).

Cell-based m^{5}C-probe methyltransferase assay

The HeLa cells were pre-incubation with various concentrations of DNA/RNA MTase inhibitors, including sinefungin, *S*-adenosyl-L-homocysteine (SAH), adenosine, and homocysteine at 37 °C for 30 min. Nine different concentrations of inhibitors were used (0.1, 0.3, 1, 3, 10, 50, 100, 250, 1000 µM) and the final DMSO concentration was kept at less than 1% v/v of the assay mix. These concentrations of inhibitors were shown to be non-toxic to HeLa cells in MTT cytotoxicity assays. This was followed by the delivery of either the m^{5}C-probe **9a** (10 µM) or control probe **10a** (10 µM) into the cells *via* Lipofectamine 2000, as described above. Aliquots were then collected for flow cytometry measurements (λ_{ex} 355 nm; λ_{em} 425-475 nm) at the indicated time points. The mean fluorescence intensity of at least 20,000 live cells was determined.

For IC$_{50}$ determination: the IC$_{50}$ value were then calculated from the variation in fluorescence at different inhibitor concentrations using nonlinear regression, with normalised dose-response fit on GraphPad Prism 6.0™. The assay was performed in triplicate for each inhibitor concentration.

For the determination of Z' factor: the Z' factor for our m^{5}C-probe assay was calculated using the method described by Zhang *et al.*[17] A total of 40 independent assays were run with either sinefungin (1 mM; positive control) or DMSO (1% v/v; negative control). The Z' factor was then calculated using the equation below.

$$Z' factor = 1 - \frac{3(\sigma p + \sigma n)}{\mu_p - \mu_n}$$

Where μ_p and μ_n are the means of the positive (p) and negative (n) controls

σ_p and σ_n are the standard deviations of the positive (p) and negative (n) controls.

The following guideline was used to interpret the Z' factor.

A Z' factor between 0.5 and 1.0 indicates an excellent assay.

A Z' factor between 0 and 0.5 indicates a marginal assay.

A Z' factor < 0 indicates too much signal overlap between the positive and negative controls for the assay to be useful.

References

1. Hudson,R.H.E. and Ghorbani-Choghamarani,A. (2007) Selective fluorometric detection of guanosine-containing sequences by 6-phenylpyrrolocytidine in DNA. *Synlett.*, **6**, 870–873.

2. Cho,S.J., Ghorbani-Choghamarani,A., Saito,Y. and Hudson,R.H.E. (2019) 6-Phenylpyrrolocytidine: an intrinsically fluorescent, environmentally responsive nucleoside analogue. *Curr. Protoc. Nucleic Acid Chem.*, **76**, e75.

3. Ren,R.X.-F., Chaudhuri,N.C., Paris,P.L., Rumney and Kool,E.T. Naphthalene, phenanthrene, and pyrene as DNA base analogues: synthesis, structure, and fluorescence in DNA. (1996) *J. Am. Chem. Soc.*, **118**, 7671-7678.

4. Yang,T., Cheong,A., Mai,X., Zou,S. and Woon, E.C.Y. (2016) A methylation-switchable conformational probe for the sensitive and selective detection of RNA demethylase activity. *Chem. Commun.*, ***52***, 6181-6184.

5. Marky,L.A. and Breslauer,K.J. (1987) Calculating thermodynamic data for transitions of any molecularity from equilibrium melting curves. *Biopolymers*, ***26***, 1601-1620.

6. Zou,S., Toh,J.D.W., Wong,K.H.Q., Gao,Y-G., Hong,W. and Woon, E.C.Y. (2016) N^6-Methyladenosine: a conformational marker that regulates the substrate specificity of human demethylases FTO and ALKBH5. *Sci. Rep.*, **6**, 25677.

7. Cheong,A., Low,J.J.A., Lim,A., Yen,P.M. and Woon,E.C.Y. (2018) A fluorescent methylation-switchable probe for highly sensitive analysis of FTO N^6-methyladenosine demethylase activity in cells. *Chem. Sci.*, **9**, 7174-7185.

8. Morris,J.V., Mahaney,M.A. and Huber,J.R. (1976) Fluorescence quantum yield determinations. 9,10-diphenylanthracene as a reference standard in different solvents. *J. Phys. Chem.*, ***80***, 969-974.

9. Williams,A.T.R. and Winfield,S.A. (1983) Relative fluorescence quantum yields using a computer-controlled luminescence spectrometer. *Analyst*, **108**, 1067-1071.

10. Wahba,A.S., Esmaeili,A., Damha,M.J. and Hudson,R.H.E. (2010) A single-label phenylpyrrolocytidine provides a molecular beacon-like response reporting HIV-1 RT RNase H activity. *Nucleic Acids Res.*, **38**, 1048-1056.

11. Netzel,T.L., Nafisi,K., Headrick,J. and Eaton,B.E. (1995) Direct observation of photoinduced electron transfer in pyrene-labeled dU nucleosides and evidence for protonated 2'-deoxyuridine anion, dU(H).cntdot., as a primary electron transfer product. *J. Phys. Chem.*, ***99***, 17948-17955.

12. Toh,J.D.W., Sun,L., Lau,L.Z.M., Tan,J., Low,J.J.A., Tang,C.W.Q., Cheong,E.J.Y., Tan,M.J.H., Chen,Y., Hong,W., Gao,Y-G. and Woon, E.C.Y. (2015) A strategy based on nucleotide specificity leads to selective inhibition of N^6-methyladenosine demethylase FTO. *Chem. Sci.*, **6**, 112-122.

13. Zhang,X., Liu,Z., Yi,J., Tang,H., Xing,J., Yu,M., Tong,T., Shang,Y., Gorospe,M. and Wang,W. (2012) NSun2 stabilizes p16INK4 mRNA by methylating the 3′-untranslated region of p16. *Nat. Commun.*, **3**, 712.

14. Wang,Z-L., Li,B., Luo,Y-X., Lin,Q., Liu,S-R., Zhang,X-Q., Zhou,H., Yang,J-H. and Qu,L-H. (2018) Comprehensive genomic characterization of RNA-binding proteins across human cancers. *Cell Rep.*, **22**, 286–298.

15. Dev,R.R., Ganji,R., Singh,S.P., Mahalingam,S., Banerjee,S. and Khosla,S. (2017). Cytosine methylation by DNMT2 facilitates stability and survival of HIV-1 RNA in the host cell during infection. *Biochem. J.*, **474**, 2009-2026.

16. Chen,Z.X., Mann,J.R., Hsieh,C.L., Riggs,A.D. and Chedin,F. *(*2005*)* Physical and functional interactions between the human DNMT3L protein and members of the de novo methyltransferase family. *J. Cell Biochem.*, **95**, *902-917.*

17. Zhang,J.H., Chung,T.D.Y. and Oldenburg,K.R. (1999) A simple statistical parameter for use in evaluation and validation of high throughput screening assays. *J. Biomol. Screen*, **4**, 67-73.
